# Population genetics and historical demographic inferences of the blue crab *Callinectes sapidus* in the US based on microsatellites

**DOI:** 10.7717/peerj.7780

**Published:** 2019-10-14

**Authors:** Danielle Macedo, Isabel Caballero, Mariana Mateos, Raphael Leblois, Shelby McCay, Luis A. Hurtado

**Affiliations:** 1 Department of Wildlife and Fisheries Sciences, Texas A&M University, College Station, TX, USA; 2 CBGP, INRA, CIRAD, IRD, Montpellier SupAgro, University of Montpellier, Montpellier, France

**Keywords:** Population genetics, Marine connectivity, Microsatellites, Crustaceans, Blue crab, Extended pelagic larval duration, Gulf of Mexico, Fisheries, Keystone species, Genetic diversity

## Abstract

The native range of the blue crab *Callinectes sapidus* spans Nova Scotia to northern Argentina. In the US, it constitutes a keystone species in estuarine habitats of the Atlantic coast and Gulf of Mexico (GOM), serving as both predator and prey to other species, and also has historically represented a multi-billion dollar fishery. Knowledge relevant to effective management and monitoring of this ecologically and economically important species, such as levels of population genetic differentiation and genetic diversity, is necessary. Although several population genetics studies have attempted to address these questions in one or more parts of its distribution, conflicting results and potential problems with the markers used, as well as other issues, have obscured our understanding on them. In this study, we examined large-scale genetic connectivity of the blue crab in the US, using 16 microsatellites, and genotyped individuals from Chesapeake Bay, in the US Atlantic, and from nine localities along the US GOM coast. Consistent with the high long-distance dispersal potential of this species, very low levels of genetic differentiation were detected for the blue crab among the ten US localities examined, suggesting it constitutes a large panmictic population within this region. Estimations of genetic diversity for the blue crab appear to be high in the US, and provide a baseline for monitoring temporal changes in this species. Demographic analyses indicate a recent range expansion of the US population, probably during the Holocene. In addition, capitalizing on published microsatellite data from southern Brazil, our analyses detected high genetic differentiation between localities in the US and Brazil. These results point to the need for examination of genetic diversity and differentiation along the area spanning the US to southern Brazil.

## Introduction

The blue crab *Callinectes sapidus* Rathbun, 1896, whose natural range spans from Nova Scotia to northern Argentina (Williams, 1984), is an estuarine keystone species that plays a crucial role in the estuarine food web as both predator and prey. Blue crabs are opportunistic foragers that feed on a variety of organisms that include other crustaceans, gastropods, fish, bivalves, algae, vascular plants, zooplankton, infauna, as well as detritus (Alexander, 1986; Fitz & Wiegert, 1991; Laughlin, 1982; Meise & Stehlik, 2003; Rosas, Lazaro-Chavez & Bückle-Ramirez, 1994). The blue crab can regulate the abundance of some of its prey populations, which can have drastic effects on the whole estuarine ecosystem (Eggleston, 1990; Mansour & Lipcius, 1991; Silliman & Bertness, 2002). As a prey, the blue crab is one of the main food items of the critically endangered whooping crane during its winter migration period in south Texas (Hunt & Slack, 1989), and reductions in the abundance of blue crabs appear to be correlated with increased mortality of whooping cranes during this period (Pugesek, Baldwin & Stehn, 2013; Stehn, 2001, 2011). Blue crabs also constitute an important prey item of the critically endangered Kemp’s Ridley sea turtle (Burke, Morreale & Standora, 1994; Seney, 2016; Witzell & Schmid, 2005), as well as of commercially important fish species, such as the red drum (Scharf & Schlicht, 2000).

The blue crab also has historically represented a multi-billion dollar fishery in the US; constituting an important economic activity in the US Middle Atlantic, South Atlantic and Gulf of Mexico (GOM) regions. In 2016 alone, 74,258 metric tons were caught in the US, worth ~$214 million (NOAA, 2018). Large local reductions of blue crabs, however, appear to have occurred in some areas, as suggested by the sharp decline in the landings of this crab in recent years, such as in the Texas coast (NOAA, 2018). Due to the ecological and commercial importance of the blue crab in the US, it is crucial to obtain information that can aid its management and monitoring, such as population genetic differentiation, genetic diversity, and demographic history (reviewed in Hauser & Carvalho, 2008).

Long-distance dispersal, and thus, low levels of genetic differentiation at large geographical scale, may be expected for the blue crab due to its extended pelagic larval duration that ranges for 4–7 weeks, followed by a postlarval megalopal stage of 1–3 weeks (Costlow & Bookhout, 1959). Thus, oceanic circulation can contribute to the dispersal of larvae and megalopae away from their parent estuaries (Epifanio, 1995; Epifanio, Valenti & Pembroke, 1984). Gene flow, however, can be affected by variation in environmental factors (e.g., salinity, temperature), as well as potential barriers for dispersal. Genetic breaks for marine species have been observed at: north vs. south of Cape Canaveral; between the Atlantic and GOM; East vs. West GOM; and between the Laguna Madre and other GOM localities (Avise, 2000; Hollenbeck, Portnoy & Gold, 2019 and references therein; Milá et al., 2017; Neigel, 2009).

Previous large-scale studies of population genetic differentiation of the blue crab in the US have been conducted using allozymes, mitochondrial restriction fragment length polymorphisms (RFLPs) and sequences, and nuclear protein-coding genes sequences. The degree of connectivity of the blue crab in its US range, however, remains uncertain, due to conflicting results among studies. Several sources of bias or lack of power (e.g., insufficient number of markers, limited genetic variability of markers, inherent limitations of markers, small sample sizes, and potential species misidentifications) could have affected one or more of these studies.

On the basis of three moderately polymorphic allozymes, a study conducted in the Texas coast reports significant spatial and temporal population genetic differences in megalopa and adult samples (Kordos & Burton, 1993). Other allozyme-based studies, however, examining a considerably higher number of allozymes, suggest panmixia within the GOM and in the US range of this species (Berthelemy-Okazaki & Okazaki, 1997; McMillen-Jackson, Bert & Steele, 1994). Moreover, genetic differences detected among megalopa populations in the Texas study could be due to misidentifications between *C. sapidus* and *C. similis* (Sullivan & Neigel, 2017). According to Sullivan & Neigel (2017), the morphological characters used by Kordos & Burton (1993) to distinguish megalopae of both species are not diagnostic. In addition, Sullivan & Neigel (2017) found a temporal composition shift in the abundance of *C. similis* and *C. sapidus* megalopae that parallels changes in the allozyme allele frequencies reported for blue crab megalopae at the same localities studied by Kordos & Burton (1993). On the other hand, inferences of genetic homogeneity in the McMillen-Jackson, Bert & Steele (1994) and Berthelemy-Okazaki & Okazaki (1997) studies may correspond to overestimates of gene flow from broad scale stabilizing selection acting at the allozyme loci surveyed (Karl & Avise, 1992).

McMillen-Jackson & Bert (2004) used RFLP analysis of the mitochondrial DNA to examine patterns of genetic variation in blue crab populations distributed from New York to the southern GOM, and found no geographic structuring. Darden (2004), however, based on variation in mitochondrial Cytochrome C Oxidase Subunit I (COI) sequences in localities along the GOM coast, reports differences between the eastern and western GOM, and among some localities within the western GOM. Based on Darden (2004), the Gulf States Marine Fisheries Commission proposed two blue crab stocks for management within the US GOM, with their division around Apalachicola, Florida (reviewed in Perry & VanderKooy, 2015). Nonetheless, a more recent study that examined variation in mitochondrial NAD2 sequences for blue crab samples collected from Massachusetts to Texas reports a lack of geographic genetic structure (Feng, Williams & Place, 2017). Mitochondrial markers, however, appear particularly problematic for inferring population connectivity and genetic diversity in the blue crab, as extremely high levels of mtDNA heteroplasmy have been recently reported in this species. Based on polymerase chain reaction (PCR), cloning, and sequencing of segments of the ND2, ND4, and COI mitochondrial loci, Williams, Feng & Place (2017) detected as many as 24 NAD2 haplotypes in a single individual (for which 17 COI haplotypes were also observed), and the dominant haplotype accounted for as little as 43.9% of the total sequences observed within an individual.

High levels of gene flow are reported in the northern GOM, between localities in the Louisiana coast and the Lower Laguna Madre, Texas, based on sequences of four nuclear protein-coding genes (Yednock & Neigel, 2014b), three of which show signatures of selection (Yednock & Neigel, 2014a). Nonetheless, significant temporal differences were found for adults between 2 years at the four genes within a single Louisiana location.

Despite being one of the most widely used markers to examine genetic connectivity in animals (Allendorf, 2017), including marine invertebrates (Selkoe et al., 2016), large-scale microsatellite-based studies are lacking for the blue crab within the US. Microsatellites usually show high levels of genetic diversity, and there is a good understanding of their use in population genetics, as well as the availability of extensive tools for their analyses (Allendorf, 2017; Selkoe & Toonen, 2006). A seemingly limited number of microsatellites, however, have been reported for the blue crab: eight highly polymorphic microsatellites (although one pair was reported in linkage disequilibrium (LD)) were developed from a blue crab individual in Chesapeake Bay, where they were shown to be highly variable (Steven et al., 2005). A set of six of these microsatellites was used to examine genetic diversity of blue crabs in Charleston Harbor, South Carolina, where they were also highly variable (Cushman & Darden, 2017). In addition, a set of seven of these microsatellites was used in a study that determined a lack of genetic differentiation for this species along a 740 Km stretch in southern Brazil (Lacerda et al., 2016). Although these microsatellites are, in general, highly diverse in the three regions where they have been used, studies of genetic differentiation in the blue crab may benefit from the addition of other polymorphic microsatellites.

Herein, we developed nine new polymorphic microsatellites for the blue crab and examined population genetic differentiation in individuals collected from nine localities along the US GOM and in the Chesapeake Bay using a total of 16 microsatellite markers. We also estimated genetic diversity and effective population size, and examined historical demography. Finally, we capitalized on a published microsatellite dataset from southern Brazil (Lacerda et al., 2016), and tested genetic differentiation and compared genetic diversity between blue crabs in that region and our study area.

## Materials and Methods

### Samples

**Figure 1:**
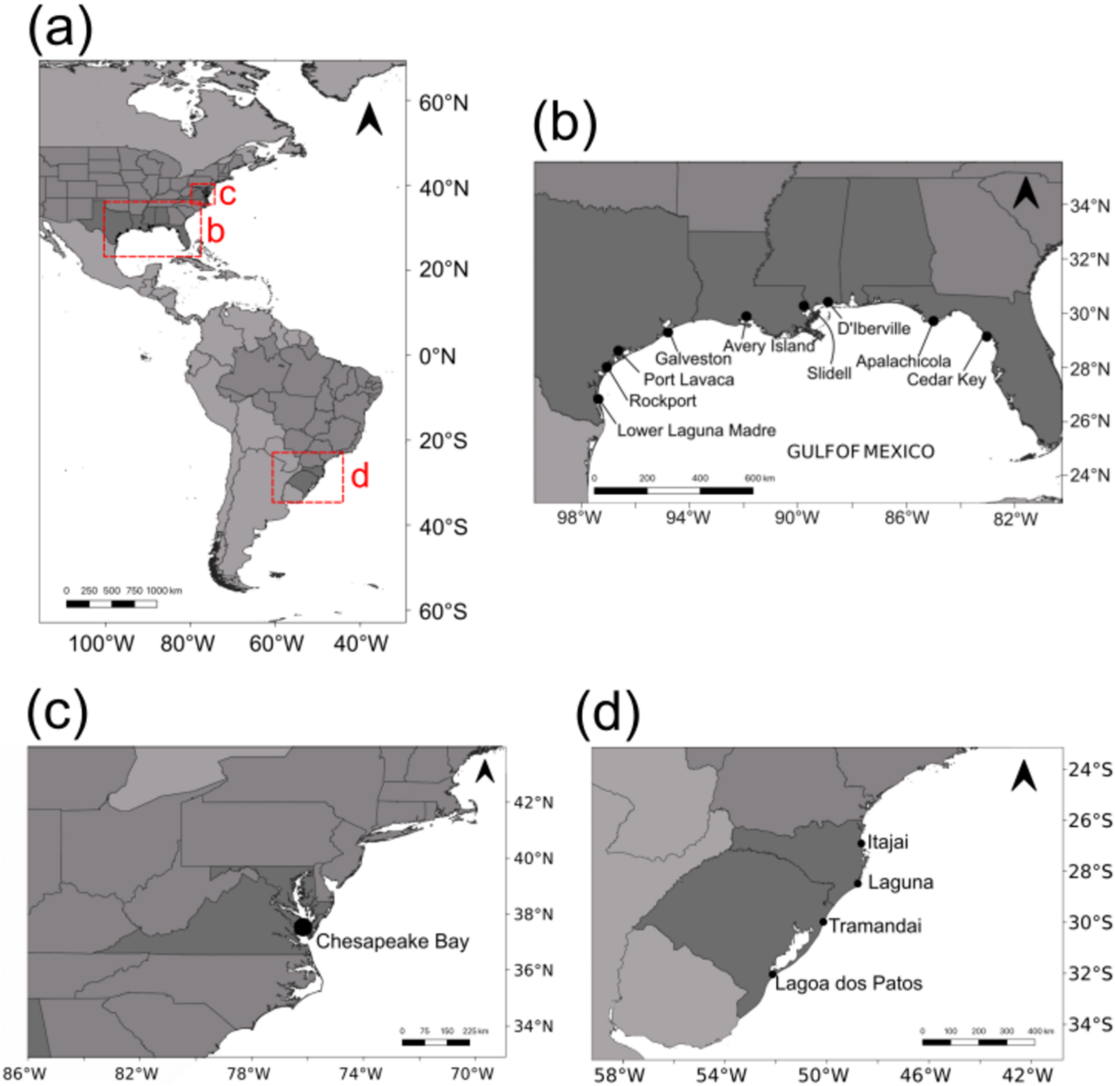
Sampling localities. (A) All sampling locations for this study (in the US) and from Lacerda et al. (2016) in southern Brazil. (B) Sampling locations in the Gulf of Mexico. (C) Sampling location in Chesapeake Bay. (D) Sampling locations from Lacerda et al. (2016) in southern Brazil.

Adult blue crabs were collected in the US from nine localities across the GOM and one in the Chesapeake Bay (Fig. 1). Specimens were assigned to *C. sapidus* on the basis of diagnostic traits (Williams, 1974, 1984). Eight localities were sampled in 2014: Rockport (ROC; *n* = 24); Port Lavaca (POL; *n* = 18); Galveston (GAL; *n* = 12); Avery Island (AVI; *n* = 20); Slidell (SLI; *n* = 11); D’Iberville (DIB; *n* = 24); Apalachicola (APA; *n* = 21); and Cedar Key (CEK; *n* = 13). Lower Laguna Madre (LLM; *n* = 24) and Chesapeake Bay (SERC; *n* = 25) were sampled in 2015. In seven GOM localities, crabs were sampled using double ring mesh nets with chicken as bait. In Rockport and D’Iberville, live crabs were purchased from local fishermen. Crabs from Chesapeake Bay were sampled by Midge Kramer (Smithsonian Environmental Research Center). Sampled crabs were stored in a cooler with dry ice, when available, or regular ice. A chela from each crab was dissected and stored in 100% ethanol for DNA preservation.

### Molecular methods

DNA was extracted from muscle tissue dissected from the chela with Quick-gDNA™ MiniPrep kit (Zymo Research Corporation, Irvine, CA, USA), according to the manufacturer’s “Solid Tissue” instructions. DNA quality was visually checked following electrophoresis on a 2% agarose gel stained with 0.1× GelRed (Biotium, Inc., Hayward, CA, USA).

For each individual, amplifications of a total of 16 microsatellites were attempted using the PCR. Nine of these (first nine in Table S1) were newly developed, whereas the remaining markers (last seven in Table S1) were reported by Steven et al. (2005) and used by Lacerda et al. (2016). PCRs were performed following the method of Schuelke (2000). For the newly developed microsatellites, an M13 universal tag sequence was added to the 5′-end of the forward primers (5′-TGTAAAACGACGGCCAGT-3′) and a seven-bp pigtail was added to the 5′-end of the reverse primers (5′-GTGTCTT-3). The addition of the pigtail forces non-template adenosine to be added to the 3′ end, thus helping reduce genotyping error (Brownstein, Carpten & Smith, 1996; Harker, 2001). The pigtail, however, was not added to the seven loci that were also used by Lacerda et al. (2016) to avoid discrepancies in allele calling between studies (see below). To insert a fluorescent dye into each reaction, a third M13 universal primer labeled with 6-FAM™, HEX™, or NED™ was added (Integrated DNA Technologies, Coralville, IA, USA; Applied Biosystems, Foster City, CA, USA).

Due to the varying degree of amplification success for each marker, different PCR reaction mixes were utilized. The seven markers (i.e., *CSA121*, *CSC094*, *CSA073*, *CSC007*, *CSC001*, *CSA035*, and *CSC004*) reported by Steven et al. (2005) and used by Lacerda et al. (2016) were amplified using the Type-It Microsatellite PCR Kit (QIAGEN, Valencia, CA, USA). Each five μL reaction contained 1× Type-It Multiplex PCR Mastermix, 1× Q Solution, 1.25 μM of the forward primer, five μM each of the reverse and M13 primers, and 40–150 ng DNA. The PCR reactions were performed on a BioRad MyCycler (Biorad, Hercules, CA, USA). They began with a denaturing step at 95 °C for 5 min, followed by 28 cycles of denaturation at 95 °C for 30 s (s), annealing at 44–58 °C for 90 s (see Table S1), and extension 72 °C for 30 s. An additional 10 cycles were used to embed the fluorescent dye, at 94 °C for 30 s, 53 °C for 45 s, and 72 °C for 45 s. A final extension at 60 °C for 30 min was used.

The nine new microsatellite loci were amplified with PCR reactions containing 40–150 ng DNA; 1× PCR buffer; 1 U *Taq* DNA polymerase (New England BioLabs, Inc., Ipswich, MA, USA); 1.25 μM forward primer; five μM of reverse and fluorescent M13 universal primer; 200 μM of each dNTP, for a final volume of 15 μL. The marker *Pen23472* had 1.6 mM MgCl_2_ added to each reaction. The thermocycler conditions consisted of: a denaturation step at 95 °C for 5 min, followed by 30 cycles of 95 °C for 30 s, 53–63 °C for 35 s (see Table S1), and extension at 72 °C for 30 s. The same 10 cycles used above were included for incorporation of the fluorescent dye. A final extension at 72 °C for 10 min was used.

Following PCR, samples were prepared for genotyping by diluting one μL of PCR products into 8.7 μL of formamide and 0.3 μL of MapMarker-ROX size standard (BioVentures, Inc., Murfreesboro, TN, USA). These were subsequently analyzed on an ABI 3130x1 Genetic Analyzer at the DNA Technologies Lab and Institute for Plant Genomics at Texas A&M University. Two researchers (DM and ICC) independently performed allele calling with GeneMarker v.1.6 (Softgenetics, State College, PA, USA) and STRand v.2.4.109 (Toonen & Hughes, 2001), respectively. Reproducibility and scoring consistency was assessed by randomly selecting 30% of the samples and repeating PCR amplification and genotyping. Negative controls (with water instead of DNA template) were included in all PCR reactions.

### Genetic analyses

#### Basic tests, genetic diversity, inbreeding and relatedness

PGDSpider v. 2.1.0.3 was used to convert data files between software packages (Lischer & Excoffier, 2012). GENEPOP on the Web 4.6 (Raymond & Rousset, 1995; Rousset, 2008) was used to test LD, estimate expected and observed heterozygosity (*H*_E_, *H*_O_), test conformity to the expectations of Hardy-Weinberg Equilibrium proportions (HWP), and calculate *F*_IS_ for each marker. To control for the occurrence of false positives due to multiple comparisons, significance of LD and HWP *p*-values was determined using: (1) the Bonferroni correction; and (2) the Benjamini–Hochberg procedure (Benjamini & Hochberg, 1995) at a false discovery rate (FDR) of 0.01 and 0.05. All GENEPOP analyses were performed with a dememorization number of 5,000, 500 batches, and 5,000 iterations per batch. Mean observed heterozygosity within populations (*H*_O_), mean expected heterozygosity within populations (*H*_S_), total heterozygosity (*H*_T_), inbreeding coefficient (*G*_IS_), allelic richness (AR), and number of alleles (*N*_A_) for each locus and group were measured with FSTAT v.2.9.3.2 (Goudet, 1995). The number of private alleles (*N*_P_) per locus was determined in GenAIEx v. 6.5 (Peakall & Smouse, 2006, 2012).

MICRO-CHECKER v. 2.2.3 (Van Oosterhout et al., 2004) was used to check for the potential presence of null alleles and scoring errors. As presence of null alleles can lead to false excess of homozygosity, and thus, overestimation of inbreeding, a Bayesian approach implemented in INEST ver. 2.1 (Chybicki & Burczyk, 2009) was used to obtain unbiased multilocus estimates of the inbreeding coefficient (*f*) while accounting for null alleles (*n*), allelic dropout (*b*) and inbreeding (*f*). INEST was initially run using the six models available, with 50,000 burn-in cycles and 500,000 cycles overall, to determine the best models for our dataset, according to the deviance information criterion (DIC). The best models were then run to 1,000,000 cycles with 100,000 burn-in cycles. Mean paired genetic relatedness values (*r*) within each locality were estimated using allele frequencies according to the mean estimate (*r*), as described by Queller & Goodnight (1989), and implemented in GenAlEx 6.5. A total of 9,999 permutations were run to generate a null distribution of *r* values on which the computed *r* was compared to assess its significance, and 10,000 bootstrap replicates were run to obtain 95% confidence intervals (CI) for each *r*.

Genetic diversity estimations obtained for US localities were compared with those obtained by Lacerda et al. (2016) for Brazil using seven loci common between the two studies. To evaluate how sample size may affect AR, a rarefaction analysis was performed in Allelic Diversity Analyzer v. 1.0 (ADZE; Szpiech, Jakobsson & Rosenberg, 2008).

Presence of loci under putative selection was tested with the method of Beaumont & Nichols (1996) implemented in the program ARLEQUIN v.3.5.2.1 (Excoffier & Lischer, 2010). This method performs coalescent simulations to estimate the distributions of heterozygosity and *F*_ST_ under the island model. Loci that do not fit neutral expectations are considered candidates of selection. Simulations assumed 100 demes with 20,000 simulated loci. Analyses were conducted considering either ten groups (i.e., each population corresponds to a group) or three groups (i.e., the Atlantic locality (SERC), the eastern GOM (APA, AVI, CEK, DIB, SLI), and the western GOM (GAL, POL, ROC, LLM)). The Bayesian simulation-based test of Beaumont & Balding (2004), implemented in the software Bayescan v.2.1 (Foll & Gaggiotti, 2008), was also used to detect the presence of loci under putative selection. This method decomposes *F*_ST_ values into locus-specific components (α) and population-specific components (β). It uses a reversible jump Markov Chain Monte Carlo (MCMC) algorithm and calculates the posterior probability that a locus is under selection by assuming two alternative models (selection-based model and neutral model). Analyses were based on 20 pilot runs, each consisting of 5,000 iterations, followed by 100,000 iterations with a burn-in of 50,000 iterations.

#### Population genetic differentiation

The statistical power of the datasets used for detecting population structure was evaluated using POWSIM v. 4.1 (Ryman & Palm, 2006). For each test, 1,000 runs/simulations were performed at four levels of population genetic differentiation and 10 generations (*t*): *F*_ST_ = 0.01 (*N*_e_ = 500, *t* = 10), *F*_ST_ = 0.007 (*N*_e_ = 750, *t* = 10), *F*_ST_ = 0.005 (*N*_e_ = 1,000, *t* = 10), and *F*_ST_ = 0.001 (*N*_e_ = 5,000, *t* = 10).

Jost’s differentiation (*D*_ST_), which quantifies relative degree of allelic differentiation, and fixation index (*G*_ST_), which quantifies nearness to fixation, were calculated using FSTAT. Population differentiation was also estimated using *F*_ST_, another measure that quantifies nearness to fixation, calculated in GenAlEx, GENODIVE (Meirmans & Van Tienderen, 2004), and FreeNA (Chapuis & Estoup, 2007). *F*_ST_ results from FreeNA were obtained with and without corrections for null alleles. FreeNA does not calculate *p*-values per se, but provides 95% CI for *F*_ST_. Therefore, CI’s that excluded zero were deemed statistically significant. *p*-values for uncorrected *F*_ST_ were estimated using Arlequin v.3.5.2.2 (Excoffier & Lischer, 2010), with 10,000 permutations and the Benjamini-Hochberg procedure; FDR values ≤ 0.05 were deemed statistically significant.

*F*_ST_ was also estimated using the private alleles method (Barton & Slatkin, 1986), as implemented in Arlequin. Recent migration rates (over the last several generations) were estimated between localities using a MCMC framework implemented in BayesAss BA3 v.3.0.4 (Wilson & Rannala, 2003). Values were estimated after 5,000,000 burnin steps to allow for convergence out of 50,000,000 iterations, sampling every 5,000 iterations. Mixing parameter values for allele frequency (-*a*), inbreeding coefficient (-*f*), and migration rate (-*m*) were set to 0.8. This value was selected after performing preliminary runs implementing different mixing parameter values (0.2 through 0.8), and examining the acceptance rates and mixing patterns of the chains (visualized in Tracer v.1.7). Multiple runs and the convergence of chains were checked by plotting traces in Tracer v.1.7. The method implemented in BayesAss assumes migration rates are relatively low and the proportion of non-migrants within a population (locality) is bound at a minimum of 2/3. Estimated non-migration rates of approximately 2/3 may indicate that the actual value is lower than this, suggesting that populations may not be distinct (BayesAss 1.3 Documentation).

Locus-by-locus analyses of molecular variance (AMOVA) and significance tests with 10,000 permutations were also performed in Arlequin. The data were grouped in multiple ways to examine patterns of variation: (1) all US localities; (2) all GOM localities; (3) GOM and Chesapeake Bay grouped separately; and (4) western and eastern GOM grouped separately.

STRUCTURE 2.2.3 (Pritchard, Stephens & Donnelly, 2000), which performs model-based clustering with a Bayesian approach, was used to examine population subdivision. *K* values from 1 to 10 were tested in three iterations, with 500,000 steps and a burn-in of 125,000 steps. Four models were used: admixture with correlated allele frequencies; admixture with independent allele frequencies; no admixture with correlated allele frequencies; and no admixture with independent allele frequencies. All other settings were set to default. Values of Ln Pr(*X*|*K*) (Pritchard, Stephens & Donnelly, 2000), Δ*K* using the Evanno method (Evanno, Regnaut & Goudet, 2005), and MedMedK, MedMeaK, MaxMedK, and MaxMeaK (Puechmaille, 2016), which are used to explore the number of *K*, were estimated using Structure Selector (Li & Liu, 2018). The LOCPRIOR setting in Structure (Hubisz et al., 2009), which is suggested in cases of weak structure, was also used to estimate clustering membership, conducting analyses for the admixture and non-admixture models with correlated allele frequencies for *K* values between 2 and 10, with five iterations for each model. The LOCPRIOR setting is useful in cases where: samples come from different populations; *F*_ST_ pairwise values between these populations are significantly different from zero; and yet results based on the default model in STRUCTURE indicate no evidence of structure. Appropriateness for using the LOCPRIOR setting was checked by assessing *r*, a parameter that estimates the informativeness of the sampling location data. Values of *r* >> 1 imply locations are non-informative about ancestry, whereas values of *r* near or below 1 imply that the ancestry proportions vary considerably between locations.

The program TESS 2.3.1 (François, Ancelet & Guillot, 2006) was also used to examine population subdivision. This program implements a Bayesian clustering algorithm for spatial population genetic studies, searching for population structure from individual multilocus genotypes sampled at distinct geographical locations without assuming predefined populations. TESS analyses were run for *K*max ranging from two to five for 50,000 sweeps, discarding the first 10,000 sweeps, and each *K* was repeated five times. A discriminant analysis of principal component analysis (DAPC) with the R package *adegenet* v.2.1.1 (Jombart, 2008; Jombart & Ahmed, 2011) also was used to examine potential population differentiation. DAPC is a non-model-based method that maximizes the differences between groups while minimizing variation within groups (Jombart, Devillard & Balloux, 2010). No prior information on population groups was assumed, and the function *find.clusters* was applied to assess the optimal number of groups based on the Bayesian information criterion method. In addition, GenAlEx was used to construct a genetic distance matrix, from which a principal coordinate analysis (PCA) was performed to identify population clusters. Finally, GENETIX v. 4.05 (Belkhir et al., 2004) was used to conduct a three-dimensional factorial correspondence analysis (FCA). This method seeks to identify correspondence between values in rows and columns, such as individuals and alleles.

Analyses of Isolation by distance (IBD) within the US and GOM were conducted using the program ISOLDE in Genepop on the Web. Two kinds of analyses were performed: (1) between localities, and (2) between individuals. Geographic distances were estimated in Google Earth Pro v.7.3 following the contours of the coastal margin between localities. Statistical significance based on the Spearman’s rank correlation coefficient was evaluated using Mantel tests (Mantel, 1967).

The aforementioned set of analyses of population genetic differentiation (except IBD) were also conducted on the genotypic data of the study by Lacerda et al. (2016) from southern Brazil, and our genotypic data including only the seven common microsatellites between both studies. Genetic diversity of blue crabs between the US and Brazil was also compared.

#### Effective population size and bottlenecks

Effective population size (*N*_e_) was estimated with NeEstimator v. 2.01 (Do et al., 2014) using the LD and heterozygote excess methods. BOTTLENECK v. 1.2.02 (Piry, Luikart & Cornuet, 1999) was used to search for signatures of a recent bottleneck (i.e., higher heterozygosity than that expected at mutation-drift equilibrium) in the blue crab populations. A Wilcoxon’s test was conducted for the three possible mutation models: infinite allele model (IAM), stepwise mutation model (SMM), and the two-phase model (TPM). For TPM, the model suggested for microsatellites, it is recommended to use 95% single-step mutations and 5% multi-step mutations, as well as a variance of 12 among multiple steps (Piry, Luikart & Cornuet, 1999). BOTTLENECK also examines the allele frequency distribution. Under mutation-drift equilibrium, an L-shaped distribution is expected, whereas a recent bottleneck is expected to cause a mode shift.

Population demographic history was also examined with MIGRAINE v0.5.1 (Rousset, Beeravolu & Leblois, 2018). Based on the population genetic differentiation results (see “Results” section), a model of a single panmictic population with a single past change in population size was assumed (OnePopVarSize, Leblois et al., 2014). One analysis was conducted with seven loci, referred to as the US-seven-loci-dataset (see “Results” section). A generalized stepwise mutation model (GSM) was used for this analysis, which reduces the risk of false positives when testing for a bottleneck. Two additional analyses were conducted with a 15-loci dataset (i.e., removing one locus that appears to be under selection; see “Results”). One of these analyses used the GSM model, and the other a combination of the GSM model for di-nucleotides and a strict SMM for tri-, tetra-, and penta-nucleotide motifs. The combined model accommodates different mutation rates for microsatellites with various motifs. For each analysis, MIGRAINE was run for a total of five iterations, each using 2,000 points, with 20,000 trees per point. Point estimates with their corresponding 95% CI were obtained for the following parameters: *pGSM*, which is the parameter of a geometric distribution determining the mutation size in number of repeats; θ_cur_ (2*N*μ), which is the current effective population size; θ_anc_ (2*N*_anc_μ), which is the ancestral effective population size; Dg/2*N*, where Dg is the time of the demographic change in generations and *N* is the effective population size; and Dg * μ, where μ is the mutation rate per generation per locus. Inferences on population contraction or expansion are based on the *N*_ratio_ (θ_cur_/θ_anc_), which is the ratio of the current effective population size divided by the ancestral one; a ratio > 1 is interpreted as a signal of population expansion, whereas a ratio < 1 as a signal of a bottleneck. Unscaled parameters of *N*, *N*_anc_, and Dg were converted using a microsatellite mutation rate of 0.0005 per locus per generation, which represents a classical average value derived from many different species (Estoup & Angers, 1998; Goldstein & Schlotterer, 1999).

## Results

### Basic tests and genetic diversity estimations

Genotyping scores for all individuals are shown in Dataset S1. Reproducibility was 100% for all the samples that were repeated (30% of the total samples). LD was not detected among loci after Bonferroni correction (*p* < 0.0005), nor with the BH FDR method (FDR ≤ 0.05). Both selection tests identified locus *Pen23472* as an outlier, suggesting it may be under putative selection (Fig. S1).

Table S2 shows genetic diversity estimations and inbreeding coefficients (*F*_IS_) per locus for each of the 10 US localities (16 loci). For comparison, we also show these data for the four Brazilian localities (from Lacerda et al., 2016). Percentage of private alleles in relation to the total number of alleles observed per locality in the US was low (calculated from values shown in Table S2), ranging from 2.6% to 5.2% (average 2.96%); whereas in Brazilian localities ranged from 4.4% to 12.7% (average 8.95%).

For the 15 putatively neutral loci (i.e., excluding *Pen23472*), no significant deviations from HWP were detected in 10 of them at any US locality after Bonferroni correction (*p* < 0.0003), whereas two loci (*Tri24376* and *Tet1886*) showed deviations of HWP in only one locality, one locus (*CSA035*) in three localities, and two loci (*CSC004* and *CSC001*) in five localities (Table S2). Using the BH FDR method with FDR ≤ 0.01, 37 tests resulted in significant deviations of HWP. No significant deviations of HWP were detected in six of the 15 putatively neutral loci: *Tet6290*, *Tet1329*, *Tet603*, *CSA121*, *Di680*, and *CSC094*. For *CSA073*, *Pen9028* and *CSC007*, significant deviations of HWP were observed in only one locality. For the remaining loci, deviations of HWP were found in two to seven localities. Using a FDR ≤ 0.05, 63 tests resulted in significant deviations of HWP. No significant deviations of HWP were suggested in *Tet6290*, *Tet1329*, *Tet603*, and *CSA121*. For *CSC094* and *CSA073*, significant deviations of HWP were found only in one locality, for *Di680* in three localities, and for the remaining loci in four to nine localities. MICRO-CHECKER did not suggest the presence of potential null alleles at any locality for *Tet6290*, *Tet1329*, *Tet603*, *Pen23472*, and *CSA121*. For *Di680* and *CSC094*, null alleles were only suggested in one locality, and for *CSA073* in two localities. For the remaining eight loci, null alleles were suggested in four to 10 populations (Table S2). Pooling data from all US localities (i.e., treating the dataset as a single panmictic population; see genetic differentiation results), no significant deviations from HWP were observed in six of the neutral loci: *Tet6290*, *Tet1329*, *Tet603*, *CSA121*, *Di680*, and *CSC094*. MICRO-CHECKER analyses with pooled data did not suggest the presence of potential null alleles in five of these loci: *Tet6290*, *Tet1329*, *Tet603*, *CSA121*, and *CSC094* (Table S3). A low percentage of null alleles was suggested for *Di680* (5%) and *CSA073* (4%).

The number of alleles per locus for the 16 loci in the US ranged from six to 47; average 18.1 (Table S3). Percentage of missing data per locus ranged from 0.0% (*Tet1329*) to 11.3% (*Tet1886*), with an overall average of 3.7% (Table S3). Average mean observed (*H*_O_) and expected heterozygosity (*H*_S_) per locus within localities was 0.59 and 0.73, respectively (Table S3). Average total expected heterozygosity (*H*_T_) per locus in the US was 0.74.

For comparison, allelic range and average alleles per locus in the Brazilian localities were 3–43 and 26.0, respectively (Table S3). These values are similar to those observed in the US localities for the seven common loci between the two studies: 7–47 allelic range and 28.1 average alleles per locus. Marked differences in *H*_S_ were observed for all but one (*CSC007*) of the loci in common between the US and southern Brazil. Average *H*_O_, *H*_S_ and *H*_T_ in Brazil was 0.54, 0.58, and 0.58, respectively (Table S3); whereas these values in the US for the seven common loci between the two studies were 0.63, 0.82, and 0.82, respectively. Rarefaction analyses conducted in ADZE suggest that the sample sizes used captured most of the allelic diversity present in both regions, ~91% for the 16 loci in the US (Fig. S2).

### Inbreeding and relatedness

Among the 15 putatively neutral loci, the lowest inbreeding coefficients were estimated for the seven loci that showed the least deviations of HWP and least presence of null alleles (i.e., *Tet6290*, *Tet1329*, *Tet603*, *CSA121*, *CSC094*, *Di680*, and *CSA073*), hereafter referred to as the “US-seven-loci-dataset” (Table S3). Because null alleles were either not detected or detected at low frequency for these loci, we do not expect inbreeding estimations for them to be largely biased (null alleles can inflate homozygosity and thus inbreeding estimations). Average *G*_IS_ for all US populations for these seven loci was 0.02. Average *G*_IS_ for the remaining eight loci was 0.34, but these values are likely upwardly biased due to the presence of null alleles (see INEST results below), which were suggested in four or more populations. *G*_IS_ estimation for the outlier locus *Pen23472* was −0.040 (Table S3). Pooling data from all US localities (i.e., treating the US dataset as a single panmictic population), average *F*_*IS*_ for the US-seven-loci-dataset is 0.015. INEST results correcting for null alleles, genotyping failures and inbreeding also indicate very low inbreeding. Pooling data from all localities for the 15 neutral loci, the best models were *nb* (null alleles and genotyping failures) and *nfb* (null alleles, inbreeding coefficients, and genotyping failures), with DIC values of 19,383.164 and 19,384.124, respectively; and their estimated inbreeding coefficients were 0 and 0.021, respectively. Of the two models, the best one was *nb* (null alleles and genotyping failures), implying that inbreeding was not important. Mean paired genetic relatedness values (*r*) within each locality were very low (Fig. S3), ranging between 0.035 in Apalachicola and −0.040 in Cedar Key (average −0.0023), and none were significant (i.e., all fell within the 95% CI).

### Population genetic differentiation within the US

Within the US, very low global *D*_ST_ (Jost’s differentiation) and *G*_ST_ (fixation index) values were obtained for each locus, with the exception of *Pen23472*, which was suggested to be under putative selection (Table S4). Global average corrected *D*_ST*′*_ (measure of allelic differentiation) and *G*_ST*′*_ (measure of fixation) values per locus for the US-seven-loci-dataset, which contains the seven loci that exhibited the least deviations of HWP and the least presence of null alleles, were 0.004 and 0.006 respectively. Global average corrected *D*_*ST′*_ and *G*_*ST′*_ values per locus for the 15 putatively neutral loci (i.e., excluding *Pen23472*) were 0.002 and 0.004 respectively. In contrast, corrected global *D*_*ST′*_ and *G*_*ST′*_ values for the outlier locus *Pen23472* were higher: 0.049 and 0.062, respectively.

Using the US-seven-loci-dataset and correcting for null alleles, average pairwise *F*_ST_ was 0.008 (estimated from values in Table 1). In this analysis, the 95% CI of four of the 45 pairwise *F*_ST_ comparisons excluded zero: Apalachicola vs. Avery Island (*F*_ST_ = 0.009; CI [0.004–0.014]); Apalachicola vs. D’Iberville (*F*_ST_ = −0.008; CI [−0.014–0.002]); Cedar Key vs. Galveston (*F*_ST_ = 0.025; CI [0.003–0.046]); and Galveston vs. Lower Laguna Madre (*F*_ST_ = 0.014; CI [0.0004–0.027]). Without correction for null alleles, average pairwise *F*_ST_ estimations was 0.006. In this analysis, three pairwise comparisons did not include zero in the 95% CI: Apalachicola vs. D’Iberville (*F*_*ST*_ = −0.008; CI [−0.014 to −0.002]); Cedar Key vs. Galveston (*F*_ST_ = 0.027; CI [0.003–0.046]); and Rockport vs. Slidell (*F*_ST_ = −0.009; CI [−0.014 to −0.005]); only the comparison SER vs. Port LaVaca was significant according to the FDR test (FDR ≤ 0.05). POWSIM indicates that this dataset has a 100% probability to detect differentiation for *F*_ST_ = 0.01; 98.7% to detect differentiation for *F*_ST_ = 0.007; 89.7% to detect differentiation for *F*_ST_ = 0.005; and 15.2% to detect differentiation for *F*_ST_ = 0.001.

**Table 1:** Pairwise *F*_ST_ values based on the US-seven-loci-dataset calculated with FreeNA. Correction for null alleles (below diagonal); without correction (above diagonal). Values in square brackets correspond to 95% confidence intervals. Significant values (i.e., 95% CI excludes zero) are in bold.

Population	APA	AVI	CEK	DIB	GAL	LLM	POL	ROC	SERC	SLI
APA	*	0.0049 [−0.0019–0.0095]	0.0124 [−0.0063–0.0370]	**−0.0083** **[−0.0140 to −0.0019]**	0.0167 [−0.0026–0.0313]	0.0008 [−0.0089–0.0122]	0.0183 [−0.0051–0.0639]	−0.0023 [−0.0098–0.0054]	−0.0057 [−0.0140–0.0028]	−0.00399 [−0.0205–0.0073]
AVI	**0.0093** **[0.0042–0.0138]**	*	0.0019 [−0.0129–0.0176]	0.0044 [−0.0086–0.0188]	0.0001 [−0.0179–0.0181]	0.0002 [−0.0035–0.0051]	0.0063 [−0.0114–0.0253]	−0.0053 [−0.0116–0.0004]	0.0026 [−0.0103–0.0196]	−0.002573 [−0.0283–0.0193]
CEK	0.0112 [−0.0078–0.0352]	0.0046 [−0.0044–0.0161]	*	0.0088 [−0.011102–0.0389]	**0.0272** **[0.0044–0.0492]**	0.0156 [−0.0069–0.0534]	0.0157 [−0.0035–0.0427]	0.0105 [−0.0116–0.0459]	0.0103 [−0.0101–0.0338]	0.015434 [−0.0086–0.0571]
DIB	**−0.0082** **[−0.0138 to −0.0019]**	0.0082 [−0.0071–0.0232]	0.0079 [−0.0111–0.0360]	*	0.0161 [−0.0025–0.0349]	0.0052 [−0.0043–0.0184]	0.0177 [−0.0038–0.0486]	−0.0063 [−0.0136–0.0011]	−0.0043 [−0.0098–0.0042]	0.001322 [−0.0097–0.0100]
GAL	0.0160 [−0.0015–0.0298]	0.0020 [−0.0116–0.0176]	**0.0245** **[0.0028–0.0455]**	0.0148 [−0.0035–0.0330]	*	0.0119 [−0.0045–0.0270]	0.0402 [−0.0124–0.1109]	0.0037 [−0.0076–0.0135]	0.0013 [−0.0162–0.0172]	−0.000272 [−0.0201–0.0175]
LLM	0.0052 [−0.0063–0.0216]	0.0040 [−0.0010–0.0111]	0.0191 [−0.0036–0.0564]	0.0083 [−0.0029–0.0248]	**0.0145** **[0.0004–0.0266]**	*	0.0169 [−0.0063–0.0567]	−0.0012 [−0.0079–0.0084]	0.0014 [−0.0061–0.0150]	−0.00716 [−0.0165–0.0019]
POL	0.0205 [−0.0019–0.0668]	0.0073 [−0.0083–0.0251]	0.0170 [−0.0023–0.0434]	0.0190 [−0.0036–0.0506]	0.0398 [−0.0124–0.1114]	0.0197 [−0.0035–0.0595]	*	0.0100 [−0.0072–0.0385]	0.0280 [−0.0129–0.1016]	0.008588 [−0.0284–0.0643]
ROC	−0.0011 [−0.0080–0.0059]	−0.0026 [−0.0089–0.0024]	0.0122 [−0.0091–0.0446]	−0.0055 [−0.0136–0.0016]	0.0051 [−0.0070–0.0154]	0.0012 [−0.0044–0.0107]	0.0121 [−0.0063–0.0401]	*	−0.0018 [−0.0122–0.0093]	**−0.00909** **[−0.0144 to −0.0048]**
SERC	−0.0051 [−0.0128–0.0032]	0.0028 [−0.0067–0.0169]	0.0094 [−0.0082–0.0293]	−0.0031 [−0.0075–0.0034]	0.0015 [−0.0142–0.0161]	0.0017 [−0.0047–0.0154]	0.0263 [−0.0120–0.0968]	−0.0016 [−0.0102–0.0078]	*	0.003735 [−0.0142–0.0185]
SLI	0.0009 [−0.0125–0.0100]	0.0004 [−0.0223–0.0205]	0.0182 [−0.0054–0.0622]	0.0046 [−0.0051–0.0142]	0.0014 [−0.0176–0.0179]	−0.0044 [−0.0144–0.0043]	0.0096 [−0.0242–0.0597]	−0.0057 [−0.0128–0.0004]	0.0042 [−0.0130–0.0187]	*

Using all the 15 putatively neutral loci and correcting for null alleles, average pairwise *F*_ST_ estimations was 0.006 (estimated from values in Table 2). In this analysis, the 95% CI of four of the 45 pairwise *F*_ST_ comparisons did not contain zero: Apalachicola vs. Cedar Key (*F*_ST_ = 0.016; CI [0.0013–0.032]); Apalachicola vs. Lower Laguna Madre (*F*_ST_ = 0.011; CI [0.0009–0.025]); Avery Island vs. Cedar Key (*F*_ST_ = 0.010; CI [0.0004–0.021]); and Cedar Key vs. Lower Laguna Madre (*F*_ST_ = 0.017; CI [0.0007–0.038]). Without correction for null alleles, average pairwise *F*_ST_ estimations was 0.003. Only the Apalachicola vs. Cedar Key comparison did not include zero in the CI (*F*_ST_ = 0.015; CI [0.0007–0.032]); and none was significant according to the FDR test (FDR ≤ 0.05). POWSIM indicates that this dataset has a 100% probability to detect differentiation for *F*_ST_ = 0.01 and 0.007; 99.6% to detect differentiation for *F*_ST_ = 0.005; and 30.4% to detect differentiation for *F*_ST_ = 0.001.

**Table 2:** Pairwise *F*_ST_ values for US localities based on the 15 putatively neutral loci dataset calculated with FreeNA. Correction for null alleles (below diagonal); without correction (above diagonal). Values in square brackets correspond to 95% confidence intervals. Significant values (i.e., 95% CI excludes zero) are in bold.

Population	APA	AVI	CEK	DIB	GAL	LLM	POL	ROC	SERC	SLI
APA	*	−0.0035 [−0.0101–0.0025]	**0.0154** **[0.0007–0.0320]**	0.0052 [−0.0071–0.0236]	0.0066 [−0.0049–0.0175]	0.0091 [−0.0014–0.0242]	0.0070 [−0.0059–0.0268]	0.0026 [−0.0037–0.0087]	0.0005 [−0.0083–0.0106]	−0.0014 [−0.0106–0.0072]
AVI	0.0015 [−0.0039 –0.0065]	*	0.0044 [−0.0089–0.0174]	0.0004 [−0.0077–0.0092]	−0.0034 [−0.0130–0.0063]	0.0006 [−0.0060–0.0072]	−0.0024 [−0.0127–0.0078]	−0.0012 [−0.0066–0.0041]	−0.0024 [−0.0116–0.0072]	−0.0014 [−0.0156–0.0121]
CEK	**0.0159** **[0.0013** **–0.0324]**	**0.0100** **[0.0004** **–0.0210]**	*	0.0107 [−0.0089–0.0386]	0.0026 [−0.0123–0.0189]	0.0148 [−0.0048–0.0403]	0.0024 [−0.0087–0.0158]	0.0074 [−0.0057–0.0230]	0.0030 [−0.0081–0.0156]	0.0142 [−0.0045–0.0371]
DIB	0.0047 [−0.0057 –0.0208]	0.0041 [−0.0033 –0.0126]	0.0106 [−0.0056 –0.0331]	*	0.0092 [−0.0034–0.0231]	−0.0019 [−0.0094–0.0055]	0.0047 [−0.0060–0.0187]	−0.0010 [−0.0085–0.0070]	0.0011 [−0.0059–0.0108]	0.0092 [−0.0048–0.0247]
GAL	0.0072 [−0.0030– 0.0174]	0.0020 [−0.0052 –0.0098]	0.0072 [−0.0064 –0.0217]	0.0086 [−0.0037 –0.0225]	*	−0.0007 [−0.0134–0.0141]	0.0086 [−0.0141–0.0417]	−0.0067 [−0.0185–0.0037]	−0.0048 [−0.0146–0.0052]	−0.0103 [−0.0251–0.0023]
LLM	**0.0109** **[0.0009** **–0.0251]**	0.0031 [−0.0021 –0.0082]	**0.0173** **[0.0007** **–0.0388]**	0.0004 [−0.0061 –0.0080]	0.0036 [−0.0077 –0.0168]	*	0.0059 [−0.0060–0.0231]	−0.0022 [−0.0082–0.0039]	0.0020 [−0.0055–0.0112]	0.0050 [−0.0062–0.0178]
POL	0.0105 [−0.0015 –0.0310]	0.0022 [−0.0062 –0.0114]	0.0073 [−0.0035 –0.0206]	0.0082 [−0.0032 –0.0231]	0.0120 [−0.0106 –0.0466]	0.0081 [−0.0040 –0.0262]	*	0.0020 [−0.0082–0.0149]	0.0103 [−0.0077–0.0405]	0.0035 [−0.0147–0.0265]
ROC	0.0014 [−0.0038 –0.0068]	0.0021 [−0.0037 –0.0097]	0.0116 [−0.0011 –0.0267]	−0.0011 [−0.0079 –0.0065]	−0.0035 [−0.0133 –0.0054]	0.0004 [−0.0040 –0.0064]	0.0053 [−0.0046 –0.0187]	*	−0.0003 [−0.0071–0.0066]	−0.0026 [−0.0132–0.0075]
SERC	0.0021 [−0.0064 –0.0125]	0.0023 [−0.0048 –0.0101]	0.0061 [−0.0041 –0.0177]	0.0025 [−0.0035 –0.0108]	−0.0018 [−0.0096 –0.0061]	0.0035 [−0.0023 –0.0113]	0.0127 [−0.0046 –0.0429]	−0.0006 [−0.0054 –0.0042]	*	0.0052 [−0.0056–0.0175]
SLI	0.0041 [−0.0048 –0.0130]	0.0081 [−0.0069 –0.0239]	0.0218 [−0.0022 –0.0539]	0.0121 [−0.0007 –0.0282]	−0.0056 [−0.0166 –0.0049]	0.0078 [−0.0039 –0.0240]	0.0090 [−0.0102 –0.0341]	0.0005 [−0.0078 –0.0088]	0.0103 [−0.0027 –0.0274]	*

Average pairwise *F*_ST_ using the private alleles method for the US-seven-loci-dataset was 0.002 and for the 15 neutral markers 0.005 (Table S5). None of the values were significant after Bonferroni correction nor with the BH FDR method (FDR ≤ 0.05). Migration analyses using BayesAss suggest that localities do not represent distinct populations, as non-migration rates of ~2/3 (~68%) were obtained within the localities for analyses of both datasets.

In an attempt to understand the idiosyncratic behavior of *Pen23472*, pairwise *F*_ST_ comparisons were conducted using only this locus. Average pairwise *F*_ST_ estimation for this locus was 0.06 (Table S6). After Bonferroni correction (*p* < 0.001), five comparisons were significant, whereas 14 comparisons were significant with the BH FDR method (FDR ≤ 0.05). The frequencies of the seven alleles observed in this locus show pronounced differences across populations, but a geographic pattern is not clear (Fig. S4).

Analyses of molecular variance analyses considering different groupings do not suggest genetic structure within the US, within the GOM, between Chesapeake Bay and the GOM, nor between the west and east Gulf (Table 3 shows results for the US-seven-loci-dataset; Table S7 for the 15-loci dataset). For the US-seven-loci-dataset, the percentage of genetic variation explained by the within individuals component is ~96.7%, by among individuals within localities ~2.8%, and among populations within each group was 0.5%. *F*-values for the genetic differentiation within the US, and within the Gulf, were −0.005. *F*-values for the genetic differentiation between Chesapeake Bay and the GOM, and between the west and east Gulf, were −0.002 and −0.0001, respectively. For the 15-loci dataset, in all cases most of the genetic variation is explained by the within individuals component (~79%), followed by the “among individuals within localities” component (~21%), and the “among populations within each group” component was very small (~0.3%). *F*-values for the genetic differentiation within the US, and within the Gulf, were 0.003. *F*-values for the genetic differentiation between Chesapeake Bay and the GOM, and between the west and east Gulf, were −0.003 and −0.0001, respectively.

**Table 3:** AMOVA results for different groupings based on the US-seven-loci-dataset.

Group	Source of variation	% Variation	F-stat	*F*-value	CI 2.5%	CI 97.5%	*P*-value
US	Within individuals	96.7	F_it	0.033	−0.036	0.081	0.012
	Among individuals within localities	2.8	F_is	0.028	−0.047	0.081	0.025
	Among populations within the U.S.	0.5	F_st	0.005	−0.001	0.014	0.999
GOM	Within individuals	96.9	F_it	0.031	−0.036	0.079	0.023
	Among individuals within localities	2.6	F_is	0.026	−0.048	0.077	0.050
	Among populations within the GOM	0.5	F_st	0.005	0.00008	0.012	0.999
GOM vs. CB	Within individuals	96.6	F_it	0.031	−0.034	0.077	0.012
	Among individuals within populations	2.8	F_is	0.028	−0.047	0.080	0.026
	Among populations within each group	0.5	F_sc	0.005	−0.0002	0.012	0.031
	Between GOM vs. CB	−0.2	F_ct	−0.002	−0.007	0.005	0.492
West vs. East GOM	Within individuals	96.9	F_it	0.031	−0.037	0.080	0.023
	Among individuals within localities	2.6	F_is	0.026	−0.045	0.077	0.050
	Among populations within each group	0.6	F_sc	0.006	−0.001	0.015	0.026
	Between west vs. east GOM	−0.1	F_ct	−0.001	−0.005	0.005	0.674

**Note:**

GOM, Gulf of Mexico; CB, Chesapeake Bay; CI, Confidence Interval.

**Figure 2:**
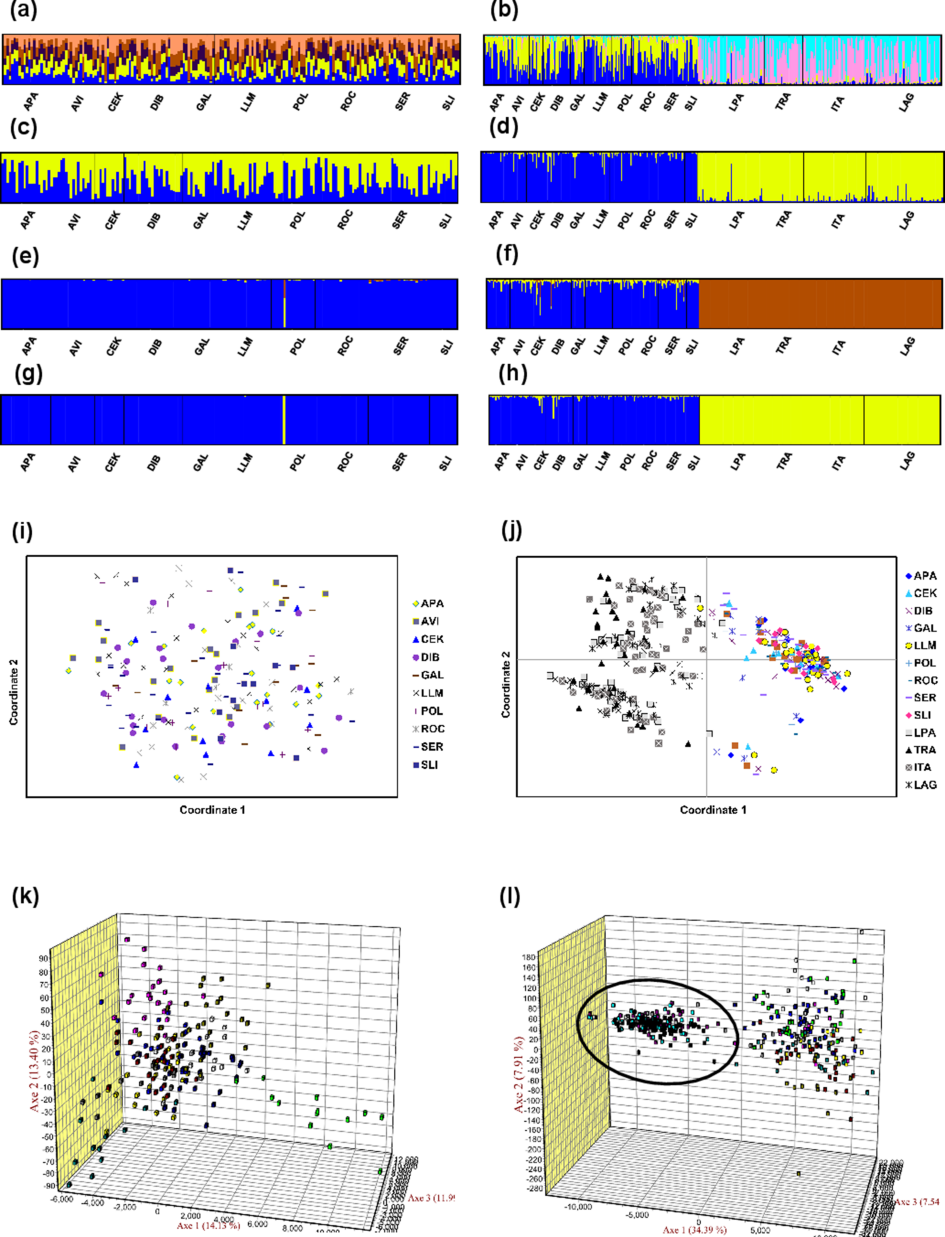
Analyses of genetic differentiation within the US (15-loci dataset) and between the US and Brazil (seven loci in common). (A and C) STRUCTURE plots for the 10 US localities using the admixture model with correlated frequencies for *K* = 5 (best *K* according to the Evanno method; A); and for *K* = 2 (C). (B and D) STRUCTURE plots for the US localities (first ten) and the Brazil localities (last four) using the non-admixture model with independent frequencies for *K* = 4 (best *K* according to the Evanno method; B); and *K* = 2 (D). (E and G) Posterior estimates of cluster membership for the 10 US localities for TESS v.2.3 using the CAR admixture model for *K*max = 3 (determined using the deviance information criterion (DIC); E) and *K*max = 2 (G). (F and H) Posterior estimates of cluster membership for the US localities (first ten) and the Brazil localities (last four) for TESS for the 10 US localities (first ten) and four localities from Brazil using the CAR admixture model for *K*max = 3 (determined using the DIC; F) and *K*max = 2 (H). Figures (A–H) were drawn using the program CLUMPAK (Kopelman et al., 2015). (I) Principal coordinate analysis (PCoA) using GENALEX for the 10 localities from the US. (J) PCoA using GENALEX for the 10 localities from the US and four localities from Brazil (gray symbols represent individuals from Brazil). (K) Factorial correspondence analysis (FCA) using GENETIX for the 10 localities from the US. (L) FCA for the 10 localities from the US and four localities from Brazil (individuals from Brazil are shown inside the oval shape).

**Figure 3:**
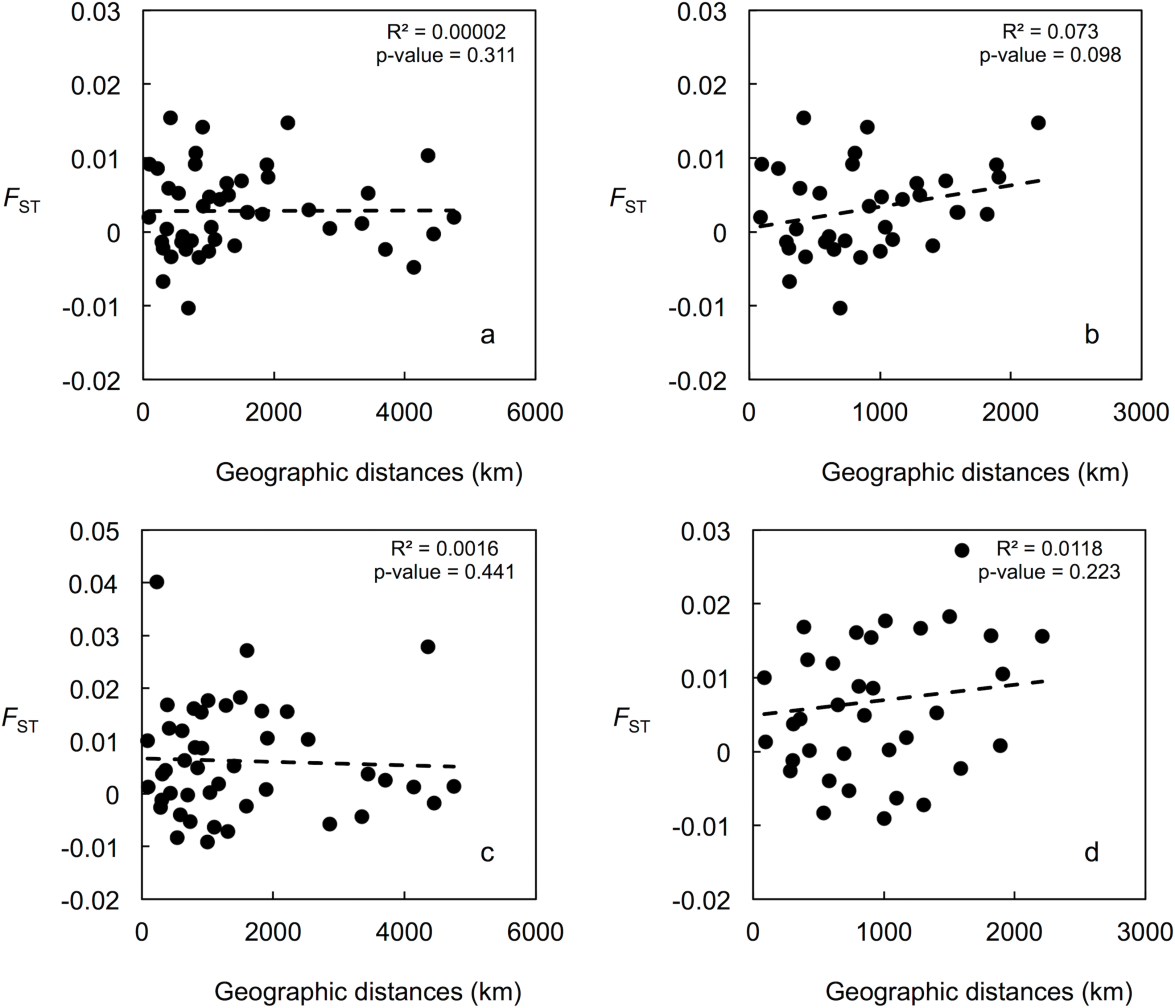
Correlation between population pairwise *F*_ST_ and geographic distance values. (A) For the 15 putatively neutral loci and all 10 US localities. (B) For the 15 putatively neutral loci and the nine Gulf of Mexico localities. (C) For the US-seven-loci-dataset and all 10 US localities. (D) For the US-seven-loci-dataset and the nine Gulf of Mexico localities.

Mean LnP (*K*) was higher for *K* = 1 in all STRUCTURE analyses. *K* values ranging from three to nine were suggested by the Evanno method, whereas the Puechmaille (2016) estimators suggest *K* = 2 in all cases. STRUCTURE plots, however, do not show evidence of population genetic structure within the US in any of the analyses (Fig. 2 shows plots for the analysis using the 15-loci dataset and the admixture model with correlated frequencies; plots for other analyses are not shown). Furthermore, no evidence of population structure was detected using the LOCPRIOR setting in Structure (plots not shown), and average values of *r* per K ranged from 6.50 to 16.75, indicating that locations are non-informative about ancestry, either because there is no population structure or the structure is independent of the locations. The other analyses for population structure, which included TESS, PCoA, FCA, and DAPC (Fig. 2; Fig. S5), also did not suggest any structure within the US. No evidence for IBD was detected within the US or GOM either conducting analyses between localities (Fig. 3) or between individuals (Fig. S6), using the US-seven-loci-dataset or the 15 putatively neutral loci.

### Genetic differentiation between US and Brazilian localities

All *F*_ST_ pairwise comparisons between US and Brazilian localities were high (range 0.11–0.21) and significant (Table S8). AMOVA defining US localities as a group and Brazilian localities as another group found also significant differences between the two regions and this differentiation explains 14.5% of the genetic variation (Table S7). Differences among populations within each group were not significant and accounted for only 0.5% of the variation. The rest of the variation was explained by differences within individuals (44.4%) and among individuals within localities (40.5%). Genetic differentiation between US and Brazil was also clearly observed in STRUCTURE, TESS, PCoA, and FCA analyses (Fig. 2).

### Effective population size estimations and demographic tests

The heterozygote excess method estimated values of *N*_e_ and 95% CI to be infinite in the US and GOM (Table S9). The LD method estimated infinite or large values for *N*_e_ (>2,000), large values for the lower limit of the 95% CI (>1,000), and infinite for the upper limit of the 95% CI. These values can be interpreted as indicative of a very large *N*_e_ (Waples & Do, 2010), but see “Discussion.” No signatures of recent bottlenecks were suggested for samples in the US with the Wilcoxon tests using the mutational models TPM (apparently the most appropriate mutational model for microsatellites) and SMM or the Mode-Shift ADT test in the BOTTLENECK program (Table S10). The Wilcoxon test using the mutational model IAM suggested signatures of recent bottlenecks for two US localities.

**Figure 4:**
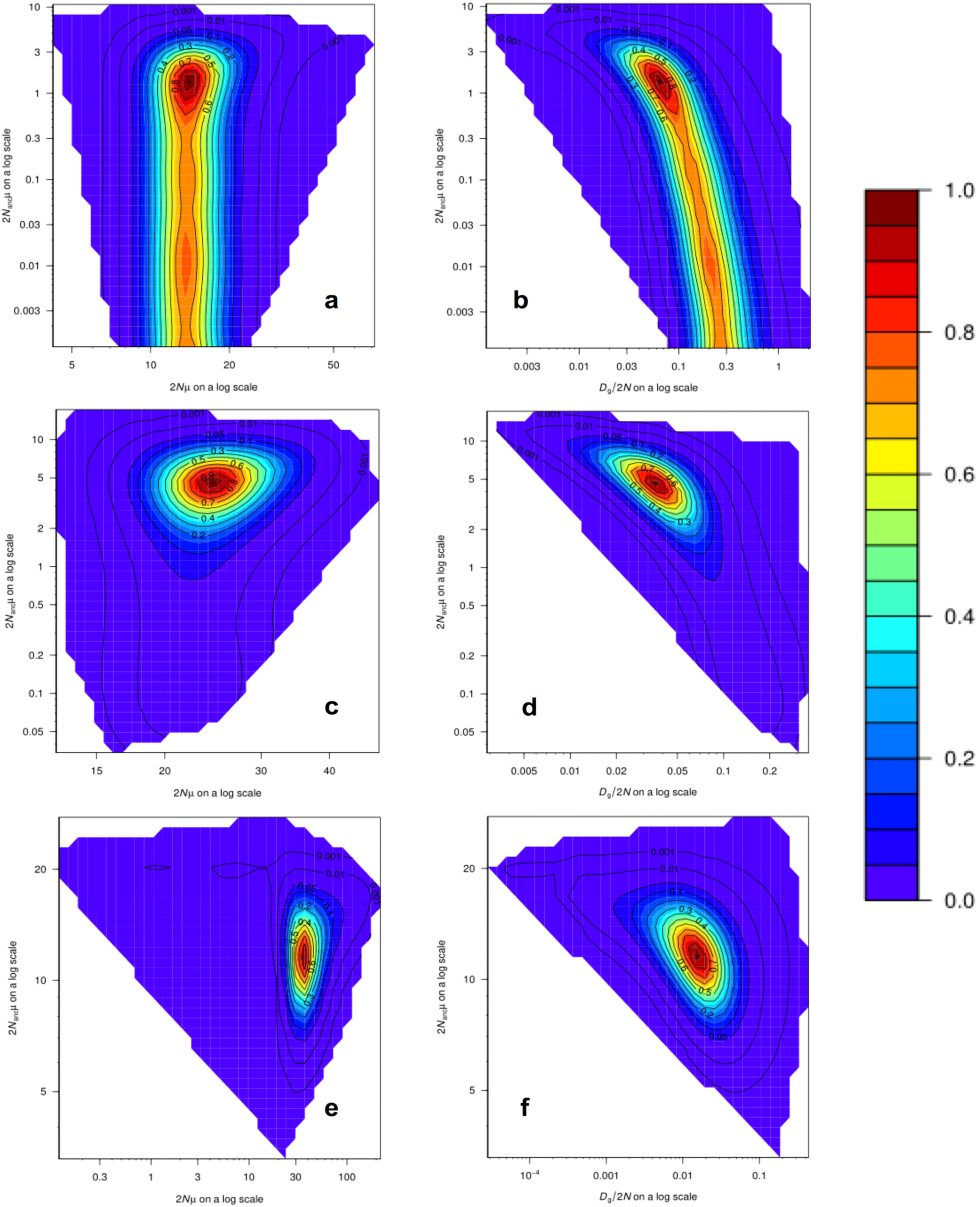
MIGRAINE pairwise likelihood ratio profiles obtained for demographic parameters. (A and B) The US-seven-loci-dataset with the GSM model. (C and D) The 15 putatively neutral loci with the GSM model. (E and F) The 15 putatively neutral loci with the SMM/GSM model. (A) (C) and (E) Ancestral effective population size 2*N*_anc_µ(θ_anc_) vs. current effective population size 2*N*µ(θ_cur_). (B) (D) and (F) Ancestral effective population size 2*N*_anc_µ(θ_anc_) vs. timing of the demographic history events *Dg*/2*N*(*D*). A very recent expansion is detected for all three models with relatively precise *D* estimates (see Table S11 for 95% CI). Effective population sizes are also relatively precise and large as shown here (one peak) for all three models (see Table S11 for 95% CI).

Analyses of past demographic history using MIGRAINE suggest an expansion of the US blue crab population (Table S11), with estimations of the current population size much larger than estimations of the ancestral effective population size, as indicated by the *N*_ratio_ (θ_cur_/θ_anc_). The *N*_ratio_ point estimate obtained for the US-seven-loci dataset using the GSM model (MaxLogLik = −483.3) was 10.27 (95% CI [2.983–19,765]). The *N*_ratio_ point estimate for the 15 loci dataset under the GSM model (MaxLogLik = −1226.7) was 5.31 (95% CI [2.48–15.44]), whereas under the combined SMM/GSM model (MaxLogLik = –1176.3) this value was 3.05 (95% CI [1.70–5.46]). Relatively precise point estimates were obtained for the different parameters, especially for the 15 loci dataset analyses, as inferred by the clear peaks in the MIGRAINE pairwise likelihood profiles (Fig. 4; see Table S11 for 95% CI). Using a mutation rate of 0.0005, the converted values of *N*, *N*_anc_ and Dg for the analysis of the 15 loci dataset with the SMM/GSM model (which had a better fit for the data) are: *N* = 17,650 (12,140–32,815) diploid individuals; Dg obtained from Dg/2*N* = 1,087 (137–6,734) generations; *N*_anc_ = 5,785 (3,798–8,400) diploid individuals; Dg obtained from Dgμ = 1,088 (322–2,764) generations.

## Discussion

This study is the first to investigate microsatellite-based population structure at a large geographic scale for the blue crab within its US range. Prior studies of this species based on a smaller number of microsatellites examined smaller areas restricted to the US Atlantic coast (Cushman & Darden, 2017; Steven et al., 2005). Our results are congruent with previous reports of substantial gene flow, and thus low levels of genetic differentiation in the blue crab among localities in the GOM or throughout its entire US distribution (Berthelemy-Okazaki & Okazaki, 1997; Feng, Williams & Place, 2017; McMillen-Jackson & Bert, 2004; McMillen-Jackson, Bert & Steele, 1994; Yednock & Neigel, 2014b).

Results of genetic differentiation were highly consistent between the dataset that excluded and the dataset that included the loci with high frequency of null alleles and hence deviations of HWP (i.e., the US-seven-loci-dataset and 15-loci-dataset, respectively). Thus, the conclusion of a lack of, or very weak, genetic differentiation for the blue crab in the US seems very robust, even in the presence of loci with null alleles. In the case of *F*_ST_ estimations, this is consistent with the prediction that although failure to correct for the presence of null alleles can lead to overestimation of *F*_ST_ when population differentiation is significant, such *F*_ST_ estimates are regarded as unbiased when population structure is absent (Chapuis & Estoup, 2007), which appears to be the case for the blue crab. *F*_ST_ estimations using the US-seven-loci dataset, which are not expected to be highly affected by null alleles, were very low with and without correcting for null alleles: average pairwise *F*_ST_ was 0.008 and 0.006, respectively. Lower values were obtained for all 15 putatively neutral loci with and without correcting for null alleles: average pairwise *F*_ST_ was 0.006 and 0.003, respectively. None of the uncorrected *F*_ST_ pairwise comparisons were significant using the 15-loci dataset, and only one was significant using the US-seven-loci-dataset, according to the FDR test (FDR ≤ 0.05). After correction for null alleles, the 95% CI of only four comparisons, in both the 15-loci and US-seven-loci datasets (albeit different pairs in each dataset analysis) excluded zero, but their lower interval values were very close to zero, and one was negative. Thus, we interpret that in general, the *F*_ST_ results indicate a lack of, or very weak, genetic differentiation for the blue crab in the US.

Similarly to *F*_ST_ estimations, *G*_ST_ (0.006 and 0.004 for the US-seven-loci-dataset and the 15 putatively neutral loci, respectively), another measure of fixation; and *D*_ST_ (0.004 and 0.002 for the US-seven-loci-dataset and the 15 putatively neutral loci, respectively), which quantifies allelic differentiation; were close to zero. Fixation and allelic differentiation measures quantify complementary aspects of population structure, although they do not necessarily correspond to each other (Jost et al., 2018). Nonetheless, *G*_ST_ approaches zero when the demes are identical in allele composition and frequencies (or when within-deme heterozygosity is high), whereas *D*_ST_ approaches zero if, and only if, all demes are identical (Jost et al., 2018). Thus, we again interpret these results as panmixia, or very weak genetic differentiation, for the blue crab in the US.

Population structure was also not detected using assignment tests (i.e., STRUCTURE and TESS), which have been shown to be highly insensitive to the presence of null alleles (Carlsson, 2008), even in species with a high null allele frequency (Rico et al., 2017). Even though in the STRUCTURE analyses the Evanno method suggests *K* values between three and nine, and the Puechmaille (2016) estimators suggest *K* = 2, mean LnP (*K*) was higher for *K* = 1 in all analyses, and STRUCTURE plots did not show evidence of any genetic structure within the US. Performance of the Evanno method has been questioned (Janes et al., 2017), whereas the Puechmaille (2016) estimators have not been tested widely, and our results suggest that they may be problematic in the case of panmictic populations. Nonetheless, Latch et al. (2006) conducted simulations of a subdivided population and found that STRUCTURE could not detect more than one population at an *F*_ST_ of 0.01, the lowest value they used. For this reason, we conducted analyses using the STRUCTURE LOCPRIOR setting, which is suggested in cases of weak structure and does not tend to find structure when none is present (see STRUCTURE Manual). These results also suggest that there is no population structure, with *r*-values much higher than the values interpreted as indicative that locations are informative. AMOVA, DAPC, PCA, FCA, and BayesAss results also did not suggest any genetic structure within the US. IBD also does not appear to be occurring for the blue crab within the US.

Conformance to HWP for pooled data at six loci is also consistent with a large panmictic population in the US. Five of these loci were not suggested to have null alleles, and the remaining one had a low percentage of potential null alleles. Null alleles could have contributed to deviations of HWP in the other loci, as they artificially inflate homozygosity. Similarly, low frequency of private alleles in the populations sampled in our study is also consistent with substantial gene flow. Average pairwise *F*_ST_ using the private alleles method was also very low (0.002 for the US-seven-loci-dataset and 0.005 for the 15 putatively neutral markers), suggesting substantial gene flow.

Remarkably similar values of genetic diversity to those reported in our study were found in a study in Charleston Harbor estuary, South Carolina (Cushman & Darden, 2017), that used six microsatellite loci, of which five overlap with our study. Both studies found very similar average number of alleles (27.8 vs. 28.8, for Charleston Harbor estuary and our study, respectively) and average expected heterozygosity (0.78 vs. 0.774, respectively) for the five loci in common. Null alleles have been shown to have weak effects on expected heterozygosity in species characterized by high prevalence of null alleles (Chapuis et al., 2008). The high similarity in genetic diversity between two independent studies is consistent with the finding of substantial gene flow across the US, suggesting that a subsample from a very small area (i.e., Charleston Harbor estuary) adequately captures the genetic diversity found in the whole region.

The South Carolina study (Cushman & Darden, 2017) reports a null allele frequency of 0 for *CSA-121*, 0.004 for *CSA-035*, 0.012 for *CSA-073*, 0.059 for *CSC-007* (this is the only marker they found deviating from HWE), and 0.093 for *CSC-094*. Pooling data across localities in our study, MICRO-CHECKER suggests a frequency of null alleles of 0.005 for *CSA-121*, 0.127 for *CSA-035*, 0.041 for *CSA-073*, 0.077 for *CSC-007* and 0.037 for *CSC-094*. Differences in the sampled range could have contributed to the observed differences between the two studies. In addition, the South Carolina study used CERVUS 3.0 (Kalinowski, Taper & Marshall, 2007) to detect null alleles, and it is reported that different methods to detect null alleles can provide different results (Dabrowski et al., 2015). Differences for *CSA-035*, however, are very marked. Although both studies observed 47 alleles for this locus, the South Carolina study reports an allelic size range of 148–256, whereas we found a range of 160–258. We sought to examine the South Carolina dataset, but unfortunately the authors of Cushman & Darden (2017) did not make it available, despite our request. Null alleles have also been reported for the southern Brazilian populations study (Lacerda et al., 2016), which used the same five loci we had in common with the South Carolina study. Null alleles are likely to occur in populations with large effective population sizes (Chapuis & Estoup, 2007), which appears to be the case for the blue crab according to our results.

Including all 16 loci, the average number of alleles per locus and expected heterozygosity for the blue crab in our study is 18.12 and 0.74, respectively. When the five microsatellites with di-motifs in our study are excluded, average number of alleles and expected heterozygosity dropped to 9.36 and is 0.64, respectively. Genetic diversity in the US was higher than in southern Brazil for the seven loci in common (three di-, four tetra-motif) between both datasets, with an average number of alleles per locus (26 vs. 28.14) and expected heterozygosity (0.58 vs. 0.82) for southern Brazil and US, respectively. Genetic diversity estimations for the blue crab in the US are also higher than those reported for the brown swimming crab *C. bellicosus* along the coast of Sonora, Gulf of California, Mexico. For this species, average number of alleles per locus, average effective number of alleles per locus, and mean observed and expected heterozygosity were 6.08, 3.9, 0.49, and 0.50, respectively (Cisneros-Mata et al., 2019).

High genetic diversity for the blue crab in the US has been also reported for mitochondrial markers (Feng, Williams & Place, 2017; McMillen-Jackson & Bert, 2004), which is in general much higher than that reported for other invertebrates, and at the upper end of other crustaceans (Feng, Williams & Place, 2017). In the GOM, Darden (2004) found 146 unique haplotypes (*n* = 213) of *C. sapidus* with 216 variable sites for a 622-bp COI fragment. For comparison, a study of *C. bellicosus* along most of its distribution in the Gulf of California and Pacific Baja California reports only 23 haplotypes (*n* = 67) with 26 variable sites for a 658-bp COI fragment (Pfeiler et al., 2005). Nonetheless, the high estimated mtDNA diversity for *C. sapidus* may be influenced by the occurrence of high levels of heteroplasmy in this species (Williams, Feng & Place, 2017). MtDNA diversity estimates could also be inflated by misidentification; a highly divergent COI sequence found by Feng, Williams & Place (2017) could belong to *C. similis* or to a highly divergent lineage of *C. sapidus* so far restricted to Brazil (Rodrigues et al., 2017). Unfortunately, this cannot be verified because neither of these studies has made its sequence data publicly available. High genetic diversity has been also reported in DNA sequences from four nuclear markers of the blue crab in Louisiana (Yednock & Neigel, 2014a). Thus, different genetic markers indicate that the blue crab harbors high levels of genetic diversity along its US range.

Inbreeding does not appear to be a problem for blue crabs in the US. Low inbreeding was estimated for individual loci in the US-seven-loci-dataset (average *G*_IS_ = 0.02). Null alleles, which can inflate inbreeding estimations, are not expected to largely bias inbreeding estimations for these loci, because they were absent, or in low frequency. A low average inbreeding value was also obtained for these loci when considered a single panmictic population (0.015). Similarly, unbiased estimations of the inbreeding coefficient for all 15 putatively neutral loci using INEST resulted in a value of 0 for the “null alleles and genotyping failures” (*nb*) model, and 0.021 for the “null alleles, inbreeding and genotyping failures” (*nfb*) model. Low mean relatedness values within localities (average = −0.0023) are consistent with low inbreeding and high gene flow. In the South Carolina study, estimated *F*_IS_ values are low (average = 0.06), which is similar to the value estimated in southern Brazil (0.056). Similarly, low *F*_IS_ values were reported in the Louisiana coast (Yednock & Neigel, 2014a). Therefore, our results and those of other studies suggest low levels of inbreeding for the blue crab in the US.

Even though our sampling did not aim to compare temporal changes within localities, the lack of genetic differentiation among different localities sampled at different times (our samples from Lower Laguna Madre and Chesapeake Bay were collected in 2015, while all others in 2014) implies temporal genetic stability. Similarly, the samples in Cushman & Darden (2017), from Charleston Harbor estuary, South Carolina, were collected in 2012 and 2013, and the number of alleles and expected heterozygosity for both years are very similar to our values. Thus, temporal genetic stability appears to occur at a large scale. Other studies, however, have reported temporal genetic differences, but they generally occurred at a single locality and may have resulted from extraordinary events that affected the genetic makeup in localized areas. Yednock & Neigel (2014a) sampled blue crabs from nine localities in the Louisiana coast in 2010, four of which were sampled again in 2011. They did not find evidence of significant geographic or temporal genetic differentiation, with the exception of one locality that showed significant allelic frequency shifts between the 2 years for the four nuclear loci they examined. They speculate that events related to the 2010 Deepwater Horizon Oil Spill may have contributed to these differences in this locality. The other localities, however, did not show allelic changes, despite also being in the coastal area affected by the oil spill (one was separated by just ~26 Km from the one that showed temporal differences). Similarly, Feng, Williams & Place (2017) found temporal genetic differences in samples collected during 5 years in the same locality in Rhode River, Chesapeake Bay. A sample collected in 2003 was different to samples collected in 2004, 2005, 2006, and 2007, which could not be distinguished from each other. They suggest that abnormal water circulation patterns and hurricane Isabel, both of which occurred in 2003, could have altered larval dispersal and juvenile recruitment.

Two previous studies are at odds with the findings of high levels of gene flow for the blue crab in the US. On the basis of three moderately polymorphic allozymes, Kordos & Burton (1993) report significant spatial and temporal population genetic differences in megalopa and adult samples in the Texas coast. Nonetheless, the use of only three informative allozyme markers, of which one or more could be under selection due to their protein-coding nature, limits the robustness of inferred patterns (Karl & Avise, 1992). Supporting this notion, another allozyme study (McMillen-Jackson, Bert & Steele, 1994), inferred levels of gene flow consistent with panmixia among blue crab populations from New York to Texas. Although these authors observed genetic patchiness on local and range-wide geographic scales, and one locus also exhibited temporal variation, they suggest that differences in pre-settlement larval patches and subsequent selection at settlement may contribute to these patterns in the presence of high gene flow. Furthermore, the genetic differences among megalopa populations detected by Kordos & Burton (1993) could be due to misidentifications, as the morphological characters they used to distinguish *C. sapidus* from *C. similis* were later deemed unreliable by Sullivan & Neigel (2017). Indeed, Sullivan & Neigel (2017) found a temporal composition shift in the abundance of *C. similis* and *C. sapidus* megalopae that parallels changes in the allozyme allele frequencies reported for blue crab megalopae at the same localities studied by Kordos & Burton (1993). Genetic differences between concurrent samples of megalopae (*n* = 32 individuals) and adults (*n* = 49) at a locality in Chesapeake Bay have also been reported by Feng, Williams & Place (2017) at two (out of four microsatellite loci). Nonetheless, these inferences could be biased by the large number of alleles per locus (49–54), compared to the examined sample sizes.

The second study that reported significant population structure for the blue crab within the US, examined mitochondrial COI sequences and found differences between the eastern and western GOM, and among some localities within the western GOM (Darden, 2004). Based on these results, the Gulf States Marine Fisheries Commission proposed two blue crab stocks for management within the US GOM, with their division around Apalachicola, Florida (reviewed in Perry & VanderKooy, 2015). The use of mitochondrial markers to infer population connectivity and genetic diversity in the blue crab, however, appears problematic due to extremely high levels of mtDNA heteroplasmy detected in this crustacean (Williams, Feng & Place, 2017). Cloning and sequencing of segments of the ND2, ND4, and COI mitochondrial loci detected as many as 24 haplotypes in a single individual and the dominant haplotype accounted for as little as 43.9% of the total sequences (Williams, Feng & Place, 2017). This may explain the extremely high mitochondrial genetic diversity observed in this crab. Our study, which included populations at both sides of this proposed division, as well as previous studies in the region (one of which also used a mitochondrial marker), did not find evidence of genetic structure within the US GOM. Yednock & Neigel (2014a), based on nuclear protein-coding genes sequences, found no population genetic differences between samples collected in Louisiana and one sample collected in the Lower Laguna Madre, Texas.

The picture emerging from independent studies using different types of markers is that the blue crab experiences high levels of gene flow in the US Atlantic and GOM region, likely corresponding to a very large panmictic population. This is remarkable considering the variation in environmental factors (e.g., salinity, temperature), potential barriers for dispersal, and genetic breaks observed for other marine species in this region, which include differentiation between the Atlantic and GOM, the East and West GOM, and differentiation between the Laguna Madre and other GOM localities (Milá et al., 2017; Neigel, 2009). A recent study (Plough, 2017) that used >9,600 single nucleotide polymorphisms (SNPs) obtained with RAD-sequencing, however, reports low but significant genetic differentiation (*F*_ST_ = 0.0103) between blue crabs collected in Panama City, Florida (GOM), and Agawam River, Massachusetts, at the northern US Atlantic (only these two US populations were included in that study). Thus, it is possible that the use of high-throughput sequencing methods could reveal low levels of genetic differentiation that may be present for this species in the US.

High levels of gene flow have also been determined for the blue crab along 740 km in southern Brazil (Lacerda et al., 2016). Despite the apparent extraordinary long distance dispersal potential of this species, there are limits to genetic homogenization across the blue crab distribution. Yednock & Neigel (2014a) report strong genetic differentiation between samples in the GOM (from Louisiana and Texas) and Venezuela. We also found strong genetic differentiation between our US samples and those from Lacerda et al. (2016) in southern Brazil. We acknowledge, however, that combining microsatellite data from different labs can be problematic (Morin et al., 2009). Nonetheless, a strong indication of genetic differentiation between the two regions that may be largely insensitive to potential bias from the combination of data from the two labs is the observation of marked differences in expected heterozygosity in six of the seven microsatellites between the US and southern Brazil. Expected heterozygosity for southern Brazil and the US is 0.59 and 0.84, respectively. This finding is congruent with the results of Rodrigues et al. (2017), which examined COI DNA sequences and reported significant differences between the two regions; although as mentioned previously, this marker is problematic for genetic structure studies because of high levels of mitochondrial heteroplasmy in this species. Similarly, the RAD-sequencing study of Plough (2017) found that two individuals from Porto Alegre, Brazil, within the region sampled by Lacerda et al. (2016), are highly differentiated from those in the two US localities he examined. A disjunct distribution for the blue crab has been suggested (Rodrigues et al., 2017; Santos & D’Incao, 2004), with a gap in northern South America (i.e., from Guyana to northern Brazil), although this needs to be verified. Future sampling efforts are needed between the US and southern Brazil to understand the limits of the populations these two regions harbor, whether other differentiated populations are present across the blue crab range, and what factors may be associated with genetic differentiation.

Estimations of *N*_e_ suggest a very large effective population size for the blue crab in the US, which is congruent with the extremely large population size inferred from the thousands of tons that are harvested each year for this species in this region. It has been suggested that precise *N*_e_ estimates using the LD method can be obtained with this method for relatively small populations (*N*_e_ < 200), and small populations are not likely to be mistaken for large ones; but it is very difficult to obtain reliable estimates for large populations with this method (Waples & Do, 2010). Estimations of *N*_e_ in the Charleston Harbor estuary using the LD method also suggest a very large population (Cushman & Darden, 2017). They obtained negative values for *N*_e_ estimations, which are interpreted as indicative of a very large *N*_e_, with lower 95% CI values ranging from 334–4,267. However, the interpretation of negative values using the LD method as indicating very large (infinite) *N*_e_ has been challenged. A recent study indicates that for medium-sized populations (one million individuals) and common sample sizes (50 individuals), negative estimates with the LD method are likely to occur, and that on average *N*_e_ estimates are negatively biased (Marandel et al., 2019). Through simulations, they found that to obtain sufficiently precise estimates of *N*_e_, ~ 1% of the total number of individuals in a population might need to be sampled. This probably corresponds to millions of individuals in the case of the blue crab US population.

Our demographic analyses suggest a recent expansion of the US blue crab population. According to the analysis of the 15 loci dataset with the SMM/GSM model, which had the best fit for the data, the population grew between 1.7 and 5.5 times. Using a mutation rate of 5 × 10^−4^, a large *N*_e_ was estimated (17,650 individuals), which is consistent with the previous analyses of *N*_e_. The expansion appears to have occurred recently, based on the point estimate for Dg (i.e., the time of the demographic change in generations) of 1,088 generations ago, and given that blue crabs live between 2 and 3 years. Considering the upper 95% CI value of Dg (6,734 generations), which was obtained from Dg/2*N*, this expansion could have occurred several thousands years ago. Although mutation rates can be very variable, changes in one order of magnitude still result in large *N*_e_ and a recent time of demographic change estimations. Assuming mutation rates of 5 × 10^−3^ and 5 × 10^−5^, conversions of *N*_e_ result in 1,765 and 176,500 individuals, respectively, and of Dg in 108.7 and 10,880 generations, respectively. The demographic expansion of the blue crab probably occurred after the end of the last glacial period (11,650 years ago), during the Holocene, when the seas became warmer. Several marine and coastal taxa also exhibit signatures consistent with range expansions since the Last Glacial Maximum (Eberl et al., 2013; Hurtado, Lee & Mateos, 2013; Jenkins, Castilho & Stevens, 2018; Marko et al., 2010). The lineage of *C. sapidus* is estimated to have diverged from its sister lineage *C. toxotes* 0.6 to 6.4 Mya (Robles et al., 2007). Fossils of *Callinectes* crabs in the US Atlantic coast have been reported from the Pleistocene and Miocene (Rathbun, 1935).

## Conclusion and future directions

Our population genetics analyses based on microsatellites indicate substantial gene flow, high genetic diversity, a large effective population size, and no indication of inbreeding or recent bottlenecks for the blue crab in its US distribution. Lack of genetic structure in the US is consistent with other studies using different types of markers that also suggest a panmictic population in this region, with rare instances of some local temporal genetic differentiation. Detection of population structure in marine organisms characterized by extremely large populations and high dispersal potential, and/or with recently diverged populations, however, may be difficult using neutral markers, and the use of non-neutral markers has been recommended (Allendorf, Hohenlohe & Luikart, 2010; Andre et al., 2011; Liu, Sun & Hurtado, 2013; Russello et al., 2012). Therefore, it is important to use genomic techniques, such as RAD-sequencing or whole genome sequencing, to study population genetic differentiation in the blue crab, which should allow identification of potential non-neutral markers. The blue crab genome is highly variable, as indicated by the hundreds of thousands of SNPs identified in a transcriptome of this species (Yednock, Sullivan & Neigel, 2015). It is also important to include samples between the US and southern Brazil, to better understand the limits of the populations these two regions harbor, and establish whether other differentiated populations are present across the blue crab range.

## Supplemental Information

10.7717/peerj.7780/supp-1 Supplemental Information 1 Supplemental tables.

10.7717/peerj.7780/supp-2 Supplemental Information 2 Supplemental figures.

10.7717/peerj.7780/supp-3 Supplemental Information 3 Genotypes dataset. Genotypes for each individual

## Additional Information and Declarations

### Data Availability

The following information was supplied regarding data availability: The raw data is available in the Supplemental File.

## References

[ref-1] Alexander SK (1986). Diet of the blue crab, *Callinectes sapidus* Rathbun, from near shore habitats of Galveston Island, Texas. Texas Journal of Science.

[ref-2] Allendorf FW (2017). Genetics and the conservation of natural populations: allozymes to genomes. Molecular Ecology.

[ref-3] Allendorf FW, Hohenlohe PA, Luikart G (2010). Genomics and the future of conservation genetics. Nature Reviews Genetics.

[ref-4] Andre C, Larsson LC, Laikre L, Bekkevold D, Brigham J, Carvalho GR, Dahlgren TG, Hutchinson WF, Mariani S, Mudde K, Ruzzante DE, Ryman N (2011). Detecting population structure in a high gene-flow species, Atlantic herring (*Clupea harengus*): direct, simultaneous evaluation of neutral vs putatively selected loci. Heredity.

[ref-5] Avise JC (2000). Phylogeography.

[ref-6] Barton NH, Slatkin M (1986). A quasi-equilibrium theory of the distribution of rare alleles in a subdivided population. Heredity.

[ref-7] Beaumont MA, Balding DJ (2004). Identifying adaptive genetic divergence among populations from genome scans. Molecular Ecology.

[ref-8] Beaumont MA, Nichols RA (1996). Evaluating loci for use in the genetic analysis of population structure. Proceedings of the Royal Society of London. Series B: Biological Sciences.

[ref-9] Belkhir K, Borsa P, Chikhi L, Raufaste N, Bonhomme F (2004). GENETIX 4. 05, Windows TM software for population genetics. Laboratoire génome, populations, interactions.

[ref-10] Benjamini Y, Hochberg Y (1995). Controlling the false discovery rate: a practical and powerful approach to multiple testing. Journal of the Royal Statistical Society: Series B (Methodological).

[ref-11] Berthelemy-Okazaki NJ, Okazaki RK (1997). Population genetics of the blue crab *Callinectes sapidus* from the Northwestern Gulf of Mexico. Gulf of Mexico Science.

[ref-12] Brownstein MJ, Carpten JD, Smith JR (1996). Modulation of non-templated nucleotide addition by Taq DNA polymerase: primer modifications that facilitate genotyping. BioTechniques.

[ref-13] Burke VJ, Morreale SJ, Standora EA (1994). Diet of the Kemp’s ridley sea turtle, *Lepidochelys kempii*, in New York waters. Fishery Bulletin.

[ref-14] Carlsson J (2008). Effects of microsatellite null alleles on assignment testing. Journal of Heredity.

[ref-15] Chapuis MP, Estoup A (2007). Microsatellite null alleles and estimation of population differentiation. Molecular Biology and Evolution.

[ref-16] Chapuis MP, Lecoq M, Michalakis Y, Loiseau A, Sword G, Piry S, Estoup A (2008). Do outbreaks affect genetic population structure? A worldwide survey in *Locusta migratoria*, a pest plagued by microsatellite null alleles. Molecular Ecology.

[ref-17] Chybicki IJ, Burczyk J (2009). Simultaneous estimation of null alleles and inbreeding coefficients. Journal of Heredity.

[ref-18] Cisneros-Mata MÁ, Munguía-Vega A, Rodríguez-Félix D, Aragón-Noriega EA, Grijalva-Chon JM, Arreola-Lizárraga JA, Hurtado LA (2019). Genetic diversity and metapopulation structure of the brown swimming crab (*Callinectes bellicosus*) along the coast of Sonora, Mexico: Implications for fisheries management. Fisheries Research.

[ref-19] Costlow JDJ, Bookhout CG (1959). The larval development of *Callinectes sapidus* Rathbun reared in the laboratory. Biological Bulletin.

[ref-20] Cushman E, Darden T (2017). Genetic characterization of Atlantic Blue Crab (*Callinectes sapidus*) in Charleston Harbor, South Carolina. Journal of Shellfish Research.

[ref-21] Dabrowski MJ, Bornelov S, Kruczyk M, Baltzer N, Komorowski J (2015). ’True’ null allele detection in microsatellite loci: a comparison of methods, assessment of difficulties and survey of possible improvements. Molecular Ecology Resources.

[ref-22] Darden RL (2004). Population genetics of the blue crab in the Gulf of Mexico.

[ref-23] Do C, Waples RS, Peel D, Macbeth GM, Tillett BJ, Ovenden JR (2014). NeEstimator v2: re-implementation of software for the estimation of contemporary effective population size (Ne) from genetic data. Molecular Ecology Resources.

[ref-24] Eberl R, Mateos M, Grosberg RK, Santamaria CA, Hurtado LA (2013). Phylogeography of the supralittoral isopod *Ligia occidentalis* around the Point Conception marine biogeographical boundary. Journal of Biogeography.

[ref-25] Eggleston DB (1990). Foraging behavior of the blue crab, *Callinectes sapidus*, on juvenile oysters, *Crassostrea virginica*: effects of prey density and size. Bulletin of Marine Science.

[ref-26] Epifanio C (1995). Transport of blue crab (*Callinectes sapidus*) larvae in the waters off mid-Atlantic states. Bulletin of Marine Science.

[ref-27] Epifanio C, Valenti C, Pembroke A (1984). Dispersal and recruitment of blue crab larvae in Delaware Bay, U.S.A. Estuarine, Coastal and Shelf Science.

[ref-28] Estoup A, Angers B, Carvalho GR (1998). Microsatellites and minisatellites for molecular ecology: theoretical and empirical considerations. Advanced Molecular Ecology.

[ref-29] Evanno G, Regnaut S, Goudet J (2005). Detecting the number of clusters of individuals using the software structure: a simulation study. Molecular Ecology.

[ref-30] Excoffier L, Lischer HEL (2010). Arlequin suite ver 3.5: a new series of programs to perform population genetics analyses under Linux and Windows. Molecular Ecology Resources.

[ref-31] Feng X, Williams EP, Place AR (2017). High genetic diversity and implications for determining population structure in the blue crab *Callinectes sapidus*. Journal of Shellfish Research.

[ref-32] Fitz HC, Wiegert RG (1991). Utilization of the intertidal zone of a salt marsh by the blue crab *Callinectes sapidus* density, return frequency, and feeding habits. Marine Ecology Progress Series.

[ref-33] Foll M, Gaggiotti O (2008). A genome-scan method to identify selected loci appropriate for both dominant and codominant markers: a Bayesian perspective. Genetics.

[ref-34] François O, Ancelet S, Guillot G (2006). Bayesian clustering using hidden Markov random fields in spatial population genetics. Genetics.

[ref-35] Goldstein DB, Schlotterer C (1999). Microsatellites: evolution and applications.

[ref-36] Goudet J (1995). FSTAT (version 1.2): a computer program to calculate F-statistics. Journal of Heredity.

[ref-37] Harker N, Henry RJ (2001). Collection, reporting and storage of microsatellite genotype data. Plant Genotyping: The DNA Fingerprinting of Plants.

[ref-38] Hauser L, Carvalho GR (2008). Paradigm shifts in marine fisheries genetics: ugly hypotheses slain by beautiful facts. Fish and Fisheries.

[ref-39] Hollenbeck CM, Portnoy DS, Gold JR (2019). Evolution of population structure in an estuarine-dependent marine fish. Ecology and Evolution.

[ref-40] Hubisz MJ, Falush D, Stephens M, Pritchard JK (2009). Inferring weak population structure with the assistance of sample group information. Molecular Ecology Resources.

[ref-41] Hunt HE, Slack RD (1989). Winter diet of whooping and sandhill cranes in south Texas. Journal of Wildlife Management.

[ref-42] Hurtado LA, Lee EJ, Mateos M (2013). Contrasting phylogeography of sandy vs. rocky supralittoral isopods in the megadiverse and geologically dynamic Gulf of California and adjacent areas. PLOS ONE.

[ref-43] Janes JK, Miller JM, Dupuis JR, Malenfant RM, Gorrell JC, Cullingham CI, Andrew RL (2017). The K = 2 conundrum. Molecular Ecology.

[ref-44] Jenkins TL, Castilho R, Stevens JR (2018). Meta-analysis of northeast Atlantic marine taxa shows contrasting phylogeographic patterns following post-LGM expansions. PeerJ.

[ref-45] Jombart T (2008). adegenet: a R package for the multivariate analysis of genetic markers. Bioinformatics.

[ref-46] Jombart T, Ahmed I (2011). adegenet 1.3-1: new tools for the analysis of genome-wide SNP data. Bioinformatics.

[ref-47] Jombart T, Devillard S, Balloux F (2010). Discriminant analysis of principal components: a new method for the analysis of genetically structured populations. BMC Genetics.

[ref-48] Jost L, Archer F, Flanagan S, Gaggiotti O, Hoban S, Latch E (2018). Differentiation measures for conservation genetics. Evolutionary Applications.

[ref-49] Kalinowski ST, Taper ML, Marshall TC (2007). Revising how the computer program cervus accommodates genotyping error increases success in paternity assignment. Molecular Ecology.

[ref-50] Karl SA, Avise JC (1992). Balancing selection at allozyme loci in oysters: implications from nuclear RFLPs. Science.

[ref-114] Kopelman NM, Mayzel J, Jakobsson M, Rosenberg NA, Mayrose I (2015). Clumpak: a program for identifying clustering modes and packaging population structure inferences across K. Molecular Ecology Resources.

[ref-51] Kordos LM, Burton RS (1993). Genetic differentiation of Texas Gulf Coast populations of the blue crab *Callinectes sapidus*. Marine Biology.

[ref-52] Lacerda ALF, Kersanach R, Cortinhas MCS, Prata PFS, Dumont LFC, Proietti MC, Maggioni R, D’Incao F (2016). High connectivity among blue crab (*Callinectes sapidus*) populations in the western south Atlantic. PLOS ONE.

[ref-53] Latch EK, Dharmarajan G, Glaubitz JC, Rhodes OE (2006). Relative performance of Bayesian clustering software for inferring population substructure and individual assignment at low levels of population differentiation. Conservation Genetics.

[ref-54] Laughlin RA (1982). Feeding habits of the blue crab, *Callinectes sapidus* Rathbun, in the Apalachicola estuary, Florida. Bulletin of Marine Science.

[ref-55] Leblois R, Pudlo P, Neron J, Bertaux F, Reddy Beeravolu C, Vitalis R, Rousset F (2014). Maximum-likelihood inference of population size contractions from microsatellite data. Molecular Biology and Evolution.

[ref-56] Li YL, Liu JX (2018). StructureSelector: a web-based software to select and visualize the optimal number of clusters using multiple methods. Molecular Ecology Resources.

[ref-57] Lischer HE, Excoffier L (2012). PGDSpider: an automated data conversion tool for connecting population genetics and genomics programs. Bioinformatics.

[ref-58] Liu S, Sun J, Hurtado LA (2013). Genetic differentiation of *Portunus trituberculatus*, the world’s largest crab fishery, among its three main fishing areas. Fisheries Research.

[ref-59] Mansour RA, Lipcius RN (1991). Density-dependent foraging and mutual interference in blue crabs preying upon infaunal clams. Marine Ecology Progress Series.

[ref-60] Mantel N (1967). The detection of disease clustering and a generalized regression approach. Cancer Research.

[ref-61] Marandel F, Lorance P, Berthelé O, Trenkel VM, Waples RS, Lamy JB (2019). Estimating effective population size of large marine populations, is it feasible?. Fish and Fisheries.

[ref-62] Marko PB, Hoffman JM, Emme SA, McGovern TM, Keever CC, Nicole Cox L (2010). The ‘expansion-contraction’model of Pleistocene biogeography: rocky shores suffer a sea change?. Molecular Ecology.

[ref-63] McMillen-Jackson AL, Bert TM (2004). Mitochondrial DNA variation and population genetic structure of the blue crab *Callinectes sapidus* in the eastern United States. Marine Biology.

[ref-64] McMillen-Jackson AL, Bert TM, Steele P (1994). Population genetics of the blue crab *Callinectes sapidus*: modest population structuring in a background of high gene flow. Marine Biology.

[ref-65] Meirmans PG, Van Tienderen PH (2004). GENOTYPE and GENODIVE: two programs for the analysis of genetic diversity of asexual organisms. Molecular Ecology Resources.

[ref-66] Meise CJ, Stehlik LL (2003). Habitat use, temporal abundance variability, and diet of blue crabs from a New Jersey estuarine system. Estuaries.

[ref-67] Milá B, Van Tassell JL, Calderón JA, Rüber L, Zardoya R (2017). Cryptic lineage divergence in marine environments: genetic differentiation at multiple spatial and temporal scales in the widespread intertidal goby *Gobiosoma bosc*. Ecology and Evolution.

[ref-68] Morin PA, Manaster C, Mesnick SL, Holland R (2009). Normalization and binning of historical and multi-source microsatellite data: overcoming the problems of allele size shift with allelogram. Molecular Ecology Resources.

[ref-69] Neigel JE (2009). Population genetics and biogeography of the Gulf of Mexico. Gulf of Mexico: Origins, Waters and Biota.

[ref-70] NOAA (2018). Landings. https://foss.nmfs.noaa.gov.

[ref-71] Peakall R, Smouse PE (2006). GENALEX 6: genetic analysis in excel. Population genetic software for teaching and research. Molecular Ecology Notes.

[ref-72] Peakall R, Smouse PE (2012). GenAlEx 6.5: genetic analysis in excel. Population genetic software for teaching and research—an update. Bioinformatics.

[ref-73] Perry HM, VanderKooy SJ (2015). The blue crab fishery of the Gulf Of Mexico, United States: a regional management plan.

[ref-74] Pfeiler E, Hurtado LA, Knowles LL, Torre-Cosio J, Bourillon-Moreno L, Marquez-Farias JF, Montemayor-Lopez G (2005). Population genetics of the swimming crab *Callinectes bellicosus* (Brachyura : Portunidae) from the eastern Pacific Ocean. Marine Biology.

[ref-75] Piry S, Luikart G, Cornuet J-M (1999). Bottleneck: a computer program for detecting recent reductions in effective population size from allele frequency data. Journal of Heredity.

[ref-76] Plough L (2017). Population genomic analysis of the blue crab *Callinectes sapidus* using genotyping-by-sequencing. Journal of Shellfish Research.

[ref-77] Pritchard JK, Stephens M, Donnelly P (2000). Inference of population structure using multilocus genotype data. Genetics.

[ref-78] Puechmaille SJ (2016). The program structure does not reliably recover the correct population structure when sampling is uneven: subsampling and new estimators alleviate the problem. Molecular Ecology Resources.

[ref-79] Pugesek BH, Baldwin MJ, Stehn T (2013). The relationship of blue crab abundance to winter mortality of Whooping Cranes. Wilson Journal of Ornithology.

[ref-80] Queller DC, Goodnight KF (1989). Estimating relatedness using genetic markers. Evolution.

[ref-81] Rathbun MJ (1935). Fossil Crustacea of the Atlantic and Gulf coastal plain. Geological Society of America, Special Paper.

[ref-82] Raymond M, Rousset F (1995). GENEPOP (version 1.2): population genetics software for exact tests and ecumenicism. Journal of Heredity.

[ref-83] Rico C, Cuesta JA, Drake P, Macpherson E, Bernatchez L, Marie AD (2017). Null alleles are ubiquitous at microsatellite loci in the Wedge Clam (*Donax trunculus*). PeerJ.

[ref-84] Robles R, Schubart CD, Conde JE, Carmona-Suárez C, Alvarez F, Villalobos JL, Felder DL (2007). Molecular phylogeny of the American *Callinectes* Stimpson, 1860 (Brachyura: Portunidae), based on two partial mitochondrial genes. Marine Biology.

[ref-85] Rodrigues MA, Dumont LFC, dos Santos CRM, D’Incao F, Weiss S, Froufe E (2017). Two distinct mtDNA lineages of the blue crab reveal large-scale population structure in its native Atlantic distribution. Estuarine, Coastal and Shelf Science.

[ref-86] Rosas C, Lazaro-Chavez E, Bückle-Ramirez F (1994). Feeding habits and food niche segregation of *Callinectes sapidus*, *C. rathbunae*, and *C. similis* in a subtropical coastal lagoon of the Gulf of Mexico. Journal of Crustacean Biology.

[ref-87] Rousset F (2008). genepop’007: a complete re-implementation of the genepop software for Windows and Linux. Molecular Ecology Resources.

[ref-88] Rousset F, Beeravolu CR, Leblois R (2018). Likelihood computation and inference of demographic and mutational parameters from population genetic data under coalescent approximations. Journal de la Société Française de Statistique.

[ref-89] Russello MA, Kirk SL, Frazer KK, Askey PJ (2012). Detection of outlier loci and their utility for fisheries management. Evolutionary Applications.

[ref-90] Ryman N, Palm S (2006). POWSIM: a computer program for assessing statistical power when testing for genetic differentiation. Molecular Ecology Resources.

[ref-91] Santos CRMdS, D’Incao F (2004). Crustáceos no cerrito Ariano Souza, Rio Grande, Rio Grande do Sul e distribuição de *Callinectes sapidus* (Brachyura, Portunidae). Iheringia, Série Zoologia, Porto Alegre.

[ref-92] Scharf FS, Schlicht KK (2000). Feeding habits of red drum (*Sciaenops ocellatus*) in Galveston Bay, Texas: seasonal diet variation and predator-prey size relationships. Estuaries.

[ref-93] Schuelke M (2000). An economic method for the fluorescent labeling of PCR fragments. Nature Biotechnology.

[ref-94] Selkoe KA, Aloia CC, Crandall ED, Iacchei M, Liggins L, Puritz JB, von der Heyden S, Toonen RJ (2016). A decade of seascape genetics: contributions to basic and applied marine connectivity. Marine Ecology Progress Series.

[ref-95] Selkoe KA, Toonen RJ (2006). Microsatellites for ecologists: a practical guide to using and evaluating microsatellite markers. Ecology Letters.

[ref-96] Seney EE (2016). Diet of Kemp’s Ridley sea turtles incidentally caught on recreational fishing gear in the northwestern Gulf of Mexico. Chelonian Conservation and Biology.

[ref-97] Silliman BR, Bertness MD (2002). A trophic cascade regulates salt marsh primary production. Proceedings of the National Academy of Sciences of the United States of America.

[ref-98] Stehn T (2001). Relationship between inflows, crabs, salinities and whooping cranes; Journey North. http://www.learner.org/jnorth/tm/crane/Stehn_CrabDocument.html.

[ref-99] Stehn T (2011). Fourth aerial census of the 2010-11 whooping crane season. http://thearansasproject.org/.

[ref-100] Steven CR, Hill J, Masters B, Place AR (2005). Genetic markers in blue crabs (*Callinectes sapidus*) I: isolation and characterization of microsatellite markers. Journal of Experimental Marine Biology and Ecology.

[ref-101] Sullivan TJ, Neigel JE (2017). Misidentification of megalopae as a potential source of error in studies of population genetics and ecology of the blue crab *Callinectes sapidus*. Marine Ecology Progress Series.

[ref-102] Szpiech ZA, Jakobsson M, Rosenberg NA (2008). ADZE: a rarefaction approach for counting alleles private to combinations of populations. Bioinformatics.

[ref-103] Toonen RJ, Hughes S (2001). Increased throughput for fragment analysis on an ABI Prism^®^ 377 automated sequencer using a membrane comb and STRand software. BioTechniques.

[ref-104] Van Oosterhout C, Hutchinson WF, Wills DP, Shipley P (2004). micro-checker: software for identifying and correcting genotyping errors in microsatellite data. Molecular Ecology Notes.

[ref-105] Waples RS, Do C (2010). Linkage disequilibrium estimates of contemporary Ne using highly variable genetic markers: a largely untapped resource for applied conservation and evolution. Evolutionary Applications.

[ref-106] Williams AB (1974). The swimming crabs of the genus *Callinectes* (Decapoda: Portunidae). Fishery Bulletin.

[ref-107] Williams AB (1984). Shrimps, lobsters, and crabs of the Atlantic coast of the eastern United States, Maine to Florida.

[ref-108] Williams EP, Feng X, Place AR (2017). Extensive heteroplasmy and evidence for fragmentation in the *Callinectes sapidus* mitochondrial genome. Journal of Shellfish Research.

[ref-109] Wilson G, Rannala B (2003). Bayesian inference of recent migration rates using multilocus genotypes. Genetics.

[ref-110] Witzell WN, Schmid JR (2005). Diet of immature Kemp’s ridley turtles (*Lepidochelys kempi*) from Gullivan Bay, Ten Thousand Islands, southwest Florida. Bulletin of Marine Science.

[ref-111] Yednock BK, Neigel JE (2014a). Detecting selection in the blue crab, *Callinectes sapidus*, using DNA sequence data from multiple nuclear protein-coding genes. PLOS ONE.

[ref-112] Yednock BK, Neigel JE (2014b). An investigation of genetic population structure in blue crabs, *Callinectes sapidus*, using nuclear gene sequences. Marine Biology.

[ref-113] Yednock BK, Sullivan TJ, Neigel JE (2015). De novo assembly of a transcriptome from juvenile blue crabs (*Callinectes sapidus*) following exposure to surrogate Macondo crude oil. BMC Genomics.

