# Supplementary material for: Population genetics and historical demographic inferences of the blue crab Callinectes sapidus in the US based on microsatellites
Source: PeerJ. 2019 Oct 14;7:e7780. doi: 10.7717/peerj.7780 (PMC6796965; doi:10.7717/peerj.7780)
Supplement: Supplemental Information 1 [file peerj-07-7780-s001.docx]

**Table SM1.** Microsatellite loci used in this study

| Locus ID | Primer Sequence (5’-3’) | Repeat Motif | Label | T_A_ |
| --- | --- | --- | --- | --- |
| Tet603 | F: TGTAAAACGACGGCCAGTGGACGAACGAAAGCCAGATA | (ACAG)_7_ | 6-FAM™ | 50°C |
|  | R: GTGTCTTCCGAGAGGAAAATGAGAAAATG |  |  |  |
| Pen9028 | F: TGTAAAACGACGGCCAGTTGTGTCCTATGTCCCAAGCA | (AAAGC)_5_ | 6-FAM™ | 58°C |
|  | R: GTGTCTTAATGGCTGTTTGCCCTACAC |  |  |  |
| Tet1057 | F: TGTAAAACGACGGCCAGTTCGCTCTCTCTCCGTTTTTC | (ATCC)_5_ | 6-FAM™ | 50°C |
|  | R: GTGTCTTCGTAGGTGGGTAGATAAGTTTGC |  |  |  |
| Tet1329 | F: TGTAAAACGACGGCCAGTCACAGCACATTACCCGTAGA | (AGAT)_7_ | 6-FAM™ | 55°C |
|  | R: GTGTCTTAAAAGGCTCGACGCCAGTAT |  |  |  |
| Pen23472 | F: TGTAAAACGACGGCCAGTTCTCTCACCCGTCTTCATCA | (AGGCG)_6_ | 6-FAM™ | 55°C |
|  | R: GTGTCTTAACTGAGGGACACACACAGC |  |  |  |
| Di680 | F: TGTAAAACGACGGCCAGTGGTCAAACAAAAAGTTCCACGA | (AC)_17_ | 6-FAM™ | 55°C |
|  | R: GTGTCTTGGGCGAACACACTTGAATAGA |  |  |  |
| Tri24376 | F: TGTAAAACGACGGCCAGTGCGGCCTACGAGAAGACTAA | (AAC)_9_ | 6-FAM™ | 58°C |
|  | R: GTGTCTTAAATGGAAAAAGACGCAACG |  |  |  |
| Tet6290 | F: TGTAAAACGACGGCCAGTTTGTCCGTGAAGTGTTCCTC | (AGAT)_16_ | 6-FAM™ | 60°C |
|  | R: GTGTCTTCCCTGATCCCTGCAAGACTA |  |  |  |
| Tet1886 | F: TGTAAAACGACGGCCAGTTTCCCAGACTTGCATAGAGTCA | (TATC)_7_ | 6-FAM™ | 57°C |
|  | R: GTGTCTTATGGCTCACAACACACCAACTA |  |  |  |
| CSC-001 | F: TGTAAAACGACGGCCAGTATTGGGTGGTTGCTTCAT | (CCTT)_14_ | 6-FAM™ | 55°C |
|  | R: ACGAGGAGAAAGTTGAGATTGC |  |  |  |
| CSC-004 | F: TGTAAAACGACGGCCAGTACAACGGTAATTGTACGAGAA | (TG)_16_ | HEX™ | 58°C |
|  | R: AGGCTAATGCCACCATCATC |  |  |  |
| CSC-007 | F: TGTAAAACGACGGCCAGTGGGACAAACAACATGAAAGTGG | (GA)_35_ | 6-FAM™ | 59°C |
|  | R: GAAAACCTATTCCGGGAAGC |  |  |  |
| CSC-094 | F: TGTAAAACGACGGCCAGTGTATCCACAACTGACTTTTCTCC | (TCTG)_6_ | HEX™ | 55°C |
|  | R: GGAGAAACACCCTCAGAAAACC |  |  |  |
| CSA-035 | F: TGTAAAACGACGGCCAGTGACTGGAGAAACGATAGGTG | (GT)_29_ | NED™ | 46°C |
|  | R: AACAAGGAGATTACACGGATTC |  |  |  |
| CSA-073 | F: TGTAAAACGACGGCCAGTGCCTATTTGCCTCGCTACCCC | (GT)_57_ | NED™ | 55°C |
|  | R: GTCACCAAAGTTGAGCAAGACTCTCT |  |  |  |
| CSA-121 | F: TGTAAAACGACGGCCAGTAATAAGAGAACAAACACACGGGG | (AGAC)_9_ | 6-FAM™ | 55°C |
|  | R: AACTGCTTGCCTTCCTTCCATC |  |  |  |

Underlined portion forward primers indicate M13 tag; underlined portion reverse primers indicate pigtail

Table SM2. Genetic diversity and inbreeding coefficient for US (16 loci) and Brazilian (seven loci) localities.

Bolded values indicate significant departures from HWP: *p* < 0.0003 for US populations (16 loci) and *p* < 0.0018 for Brazilian populations (7 loci). Underlined loci indicate significant departures from HWP using the Benjamini-Hochberg correction for multiple tests with FDR = 0.05. Loci with asterisk (*) indicate significant departures from HWP using the Benjamini-Hochberg correction for multiple tests with FDR = 0.01. Italicized loci indicate presence of null alleles is suggested. *N* = number of samples genotyped; *N_A_/N_P_* = number of alleles and private alleles; *AR* = allelic richness; *H_O_* = observed heterozygosity*; H_E_* = expected heterozygosity; *P_HW_* = probability of HWP; *F_IS_* = inbreeding coefficient.

| Apalachicola, Florida | | | | | | | | Avery Island, Louisiana | | | | | | | |
| --- | --- | --- | --- | --- | --- | --- | --- | --- | --- | --- | --- | --- | --- | --- | --- |
| Primer | N | N_A_/N_P_ | AR | H_O_ | H_E_ | P_HW_ | F_IS_ | Primer | N | N_A_/N_P_ | AR | H_O_ | H_E_ | P_HW_ | F_IS_ |
| Tet6290 | 20 | 3/1 | 2.29 | 0.250 | 0.296 | 0.4687 | 0.181 | Tet6290 | 17 | 4/0 | 2.734 | 0.294 | 0.265 | 1.0000 | -0.081 |
| Tet1329 | 20 | 3/0 | 2.58 | 0.600 | 0.466 | 0.3294 | -0.263 | Tet1329 | 18 | 3/0 | 2.389 | 0.500 | 0.508 | 1.0000 | 0.044 |
| CSA121 | 18 | 4/0 | 2.656 | 0.278 | 0.252 | 1.0000 | -0.076 | CSA121 | 18 | 3/0 | 2.351 | 0.278 | 0.323 | 0.5115 | 0.167 |
| Tet603 | 19 | 7/0 | 4.694 | 0.684 | 0.641 | 0.6875 | -0.04 | Tet603 | 18 | 6/0 | 5.067 | 1.000 | 0.724 | 0.3032 | -0.357 |
| Di680 | 20 | 14/0 | 8.709 | 0.850 | 0.888 | 0.1862 | 0.068 | Di680 | 18 | 14/1 | 9.269 | 0.889 | 0.907 | 0.1978 | 0.049 |
| CSC094 | 19 | 5/0 | 3.591 | 0.579 | 0.598 | 0.8000 | 0.059 | *CSC094* | 17 | 6/0 | 4.463 | 0.412 | 0.702 | 0.0049 | 0.439 |
| CSA073 | 20 | 17/2 | 9.283 | 0.950 | 0.900 | 0.9148 | -0.03 | CSA073 | 18 | 21/2 | 10.997 | 0.889 | 0.931 | 0.0921 | 0.073 |
| Tri24376 | 20 | 6/0 | 4.275 | 0.700 | 0.723 | 0.7855 | 0.057 | *Tri24376* | 17 | 6/0 | 5.16 | 0.412 | 0.766 | 0.0015* | 0.486 |
| *Tet1886* | 20 | 6/1 | 4.513 | 0.450 | 0.728 | 0.0162 | 0.403 | Tet1886 | 13 | 4/0 | 3.882 | 0.462 | 0.695 | 0.1982 | 0.371 |
| *Tet1057* | 20 | 4/0 | 3.399 | 0.350 | 0.525 | 0.0271 | 0.356 | *Tet1057* | 17 | 5/0 | 4.472 | 0.353 | 0.680 | 0.0012* | 0.504 |
| Pen9028 | 20 | 7/0 | 4.737 | 0.550 | 0.580 | 0.4339 | 0.077 | Pen9028 | 17 | 7/0 | 5.187 | 0.588 | 0.649 | 0.3690 | 0.123 |
| CSC007 | 19 | 16/0 | 9.883 | 0.895 | 0.920 | 0.6334 | 0.054 | CSC007 | 18 | 17/1 | 9.71 | 0.944 | 0.907 | 0.4102 | -0.012 |
| *CSC001* | 18 | 12/0 | 8.563 | 0.500 | 0.881 | **0.0000*** | 0.456 | *CSC001* | 15 | 10/0 | 8.031 | 0.267 | 0.880 | **0.0000*** | 0.714 |
| *CSA035* | 20 | 23/0 | 10.55 | 0.700 | 0.919 | 0.0016* | 0.262 | *CSA035* | 18 | 20/3 | 10.547 | 0.667 | 0.923 | **0.0000*** | 0.304 |
| *CSC004* | 18 | 20/0 | 10.972 | 0.722 | 0.934 | 0.0010 | 0.253 | *CSC004* | 17 | 16/0 | 9.385 | 0.706 | 0.893 | 0.0244 | 0.238 |
| Pen23472 | 20 | 6/0 | 4.262 | 0.800 | 0.710 | 0.0024* | -0.101 | Pen23472 | 14 | 5/0 | 4.469 | 0.857 | 0.712 | 0.0018* | -0.169 |
| Cedar Key, Florida | | | | | | | | D’Iberville, Mississippi | | | | | | | |
| Primer | N | N_A_/N_P_ | AR | H_O_ | H_E_ | P_HW_ | F_IS_ | Primer | N | N_A_/N_P_ | AR | H_O_ | H_E_ | P_HW_ | F_IS_ |
| Tet6290 | 12 | 4/0 | 3.401 | 0.500 | 0.462 | 0.3644 | -0.039 | Tet6290 | 23 | 3/0 | 2.458 | 0.391 | 0.328 | 1.0000 | -0.172 |
| Tet1329 | 12 | 3/0 | 2.98 | 0.750 | 0.594 | 0.3979 | -0.222 | Tet1329 | 24 | 4/0 | 2.582 | 0.500 | 0.497 | 0.3582 | 0.016 |
| CSA121 | 11 | 4/1 | 2.909 | 0.273 | 0.248 | 1.0000 | -0.053 | CSA121 | 23 | 3/0 | 2.347 | 0.261 | 0.235 | 1.0000 | -0.086 |
| Tet603 | 9 | 5/0 | 4.333 | 0.444 | 0.630 | 0.2683 | 0.347 | Tet603 | 24 | 8/0 | 5.055 | 0.750 | 0.662 | 0.6357 | -0.111 |
| Di680 | 12 | 11/0 | 8.9 | 0.917 | 0.896 | 0.4102 | 0.016 | Di680 | 24 | 12/0 | 7.736 | 0.875 | 0.872 | 0.8904 | 0.017 |
| CSC094 | 12 | 6/0 | 4.685 | 0.667 | 0.642 | 0.8035 | 0.006 | CSC094 | 23 | 4/0 | 3.536 | 0.565 | 0.551 | 0.9328 | -0.004 |
| CSA073 | 9 | 15/2 | 12.067 | 0.889 | 0.920 | 0.2283 | 0.092 | CSA073 | 23 | 23/0 | 10.509 | 0.913 | 0.928 | 0.0651 | 0.039 |
| *Tri24376* | 12 | 5/0 | 4.384 | 0.417 | 0.691 | 0.0191 | 0.577 | *Tri24376* | 24 | 6/0 | 4.184 | 0.458 | 0.684 | 0.0179 | 0.349 |
| Tet1886 | 12 | 6/1 | 5.282 | 0.583 | 0.722 | 0.1534 | 0.234 | Tet1886 | 22 | 6/0 | 3.713 | 0.409 | 0.571 | 0.0043 | 0.305 |
| *Tet1057* | 12 | 4/0 | 3.973 | 0.333 | 0.726 | 0.0027 | 0.571 | Tet1057 | 21 | 4/0 | 3.808 | 0.524 | 0.670 | 0.1048 | 0.241 |
| *Pen9028* | 12 | 7/0 | 6.034 | 0.500 | 0.792 | 0.0025 | 0.405 | *Pen9028* | 24 | 7/0 | 5 | 0.417 | 0.694 | 0.0092 | 0.418 |
| CSC007 | 12 | 12/0 | 8.677 | 0.833 | 0.868 | 0.2900 | 0.083 | *CSC007* | 24 | 20/1 | 10.351 | 0.750 | 0.931 | 0.0022 | 0.215 |
| *CSC001* | 11 | 9/0 | 7.759 | 0.545 | 0.864 | 0.0044 | 0.409 | *CSC001* | 23 | 14/0 | 8.578 | 0.609 | 0.890 | 0.0044 | 0.336 |
| *CSA035* | 12 | 14/0 | 10.103 | 0.500 | 0.910 | **0.0000*** | 0.484 | *CSA035* | 23 | 24/1 | 11.099 | 0.739 | 0.941 | 0.0024 | 0.236 |
| *CSC004* | 12 | 16/0 | 11.362 | 0.667 | 0.931 | 0.0014 | 0.323 | CSC004 | 24 | 20/0 | 9.89 | 0.917 | 0.913 | 0.6982 | 0.017 |
| Pen23472 | 12 | 4/0 | 4.542 | 0.917 | 0.719 | 0.0129 | -0.198 | Pen23472 | 24 | 7/0 | 4.648 | 0.667 | 0.714 | 0.3914 | 0.087 |

Table SM2 (continued)

| Galveston, Texas | | | | | | | | Lower Laguna Madre, Texas | | | | | | | |
| --- | --- | --- | --- | --- | --- | --- | --- | --- | --- | --- | --- | --- | --- | --- | --- |
| Primer | N | N_A_/N_P_ | AR | H_O_ | H_E_ | P_HW_ | F_IS_ | Primer | N | N_A_/N_P_ | AR | H_O_ | H_E_ | P_HW_ | F_IS_ |
| Tet6290 | 12 | 3/0 | 2.42 | 0.250 | 0.226 | 1.0000 | -0.065 | Tet6290 | 22 | 3/0 | 1.636 | 0.091 | 0.088 | 1.0000 | -0.012 |
| Tet1329 | 12 | 2/0 | 1.994 | 0.417 | 0.330 | 1.0000 | -0.222 | Tet1329 | 24 | 5/2 | 3.233 | 0.667 | 0.531 | 0.5923 | -0.235 |
| CSA121 | 12 | 3/0 | 2.564 | 0.333 | 0.344 | 0.1811 | 0.074 | CSA121 | 24 | 5/1 | 3.469 | 0.333 | 0.444 | 0.0492 | 0.27 |
| Tet603 | 12 | 8/1 | 6.305 | 0.833 | 0.740 | 0.9753 | -0.084 | Tet603 | 24 | 9/0 | 4.902 | 0.625 | 0.636 | 0.2464 | 0.039 |
| Di680 | 12 | 10/0 | 7.615 | 0.750 | 0.840 | 0.0154 | 0.15 | Di680 | 20 | 16/0 | 9.494 | 0.950 | 0.913 | 0.5344 | -0.015 |
| CSC094 | 12 | 4/0 | 3.817 | 0.667 | 0.681 | 0.6627 | 0.064 | CSC094 | 24 | 5/0 | 4.008 | 0.708 | 0.623 | 0.3378 | -0.116 |
| CSA073 | 11 | 11/0 | 8.389 | 0.727 | 0.868 | 0.2193 | 0.208 | *CSA073* | 19 | 17/1 | 10.019 | 0.737 | 0.922 | 0.0025* | 0.227 |
| Tri24376 | 12 | 5/1 | 4.145 | 0.417 | 0.656 | 0.0283 | 0.402 | *Tri24376* | 19 | 7/1 | 4.421 | 0.263 | 0.679 | **0.0000*** | 0.629 |
| *Tet1886* | 12 | 6/0 | 5.34 | 0.333 | 0.774 | 0.0023 | 0.598 | *Tet1886* | 24 | 4/0 | 3.052 | 0.375 | 0.565 | 0.0553 | 0.355 |
| *Tet1057* | 12 | 4/0 | 3.654 | 0.250 | 0.514 | 0.0158 | 0.545 | *Tet1057* | 24 | 5/0 | 4.314 | 0.417 | 0.690 | 0.0036* | 0.414 |
| Pen9028 | 12 | 7/0 | 6.119 | 0.667 | 0.785 | 0.0978 | 0.193 | Pen9028 | 24 | 8/0 | 5.603 | 0.625 | 0.759 | 0.0946 | 0.197 |
| CSC007 | 12 | 14/0 | 9.889 | 0.750 | 0.903 | 0.0722 | 0.211 | CSC007 | 24 | 22/2 | 10.311 | 0.833 | 0.927 | 0.0915 | 0.122 |
| CSC001 | 12 | 10/0 | 7.525 | 0.667 | 0.833 | 0.0427 | 0.241 | *CSC001* | 21 | 11/0 | 7.511 | 0.429 | 0.858 | **0.0000*** | 0.519 |
| *CSA035* | 12 | 17/0 | 11.392 | 0.750 | 0.927 | 0.0054 | 0.233 | *CSA035* | 23 | 23/1 | 10.978 | 0.826 | 0.940 | 0.0629 | 0.143 |
| *CSC004* | 12 | 16/0 | 11.212 | 0.500 | 0.927 | **0.0000*** | 0.494 | *CSC004* | 24 | 19/0 | 9.23 | 0.542 | 0.896 | **0.0000*** | 0.413 |
| Pen23472 | 12 | 5/0 | 4.543 | 0.750 | 0.747 | 0.0045 | 0.039 | Pen23472 | 24 | 6/0 | 5.006 | 0.792 | 0.766 | 0.0003* | -0.012 |
| Port Lavaca, Texas | | | | | | | | Rockport, Texas | | | | | | | |
| Primer | N | N_A_/N_P_ | AR | H_O_ | H_E_ | P_HW_ | F_IS_ | Primer | N | N_A_/N_P_ | AR | H_O_ | H_E_ | P_HW_ | F_IS_ |
| Tet6290 | 18 | 2/0 | 1.876 | 0.222 | 0.198 | 1.0000 | -0.097 | Tet6290 | 22 | 2/0 | 1.947 | 0.318 | 0.268 | 1.0000 | -0.167 |
| Tet1329 | 18 | 4/0 | 3.266 | 0.611 | 0.574 | 0.4517 | -0.036 | Tet1329 | 22 | 3/0 | 2.539 | 0.636 | 0.505 | 0.4268 | -0.238 |
| CSA121 | 18 | 4/0 | 2.952 | 0.444 | 0.372 | 1.0000 | -0.167 | CSA121 | 22 | 3/0 | 2.738 | 0.455 | 0.377 | 1.0000 | -0.183 |
| Tet603 | 18 | 6/0 | 4.825 | 0.778 | 0.681 | 0.2590 | -0.115 | Tet603 | 22 | 6/0 | 4.512 | 0.682 | 0.630 | 0.5676 | -0.059 |
| Di680 | 18 | 14/0 | 8.901 | 0.778 | 0.890 | 0.1753 | 0.155 | *Di680* | 22 | 18/1 | 9.803 | 0.727 | 0.919 | 0.0103 | 0.231 |
| CSC094 | 17 | 5/0 | 4.277 | 0.588 | 0.721 | 0.4411 | 0.214 | CSC094 | 22 | 5/0 | 3.973 | 0.727 | 0.629 | 0.5445 | -0.133 |
| *CSA073* | 17 | 19/0 | 10.896 | 0.824 | 0.933 | 0.0657 | 0.147 | CSA073 | 22 | 21/0 | 10.442 | 0.864 | 0.930 | 0.1188 | 0.094 |
| *Tri24376* | 18 | 6/0 | 4.647 | 0.500 | 0.742 | 0.0147 | 0.352 | Tri24376 | 22 | 4/0 | 3.48 | 0.500 | 0.613 | 0.2377 | 0.206 |
| *Tet1886* | 17 | 7/0 | 5.833 | 0.353 | 0.789 | 0.0005* | 0.573 | *Tet1886* | 18 | 6/0 | 4.858 | 0.278 | 0.752 | **0.0001*** | 0.647 |
| *Tet1057* | 18 | 4/0 | 3.795 | 0.278 | 0.640 | 0.0012* | 0.585 | *Tet1057* | 20 | 5/0 | 3.927 | 0.350 | 0.565 | 0.0091 | 0.402 |
| *Pen9028* | 18 | 8/0 | 5.602 | 0.556 | 0.731 | 0.1440 | 0.267 | Pen9028 | 22 | 9/1 | 6.071 | 0.682 | 0.771 | 0.2337 | 0.138 |
| *CSC007* | 17 | 20/1 | 11.16 | 0.765 | 0.936 | 0.0071 | 0.212 | *CSC007* | 22 | 18/0 | 9.74 | 0.773 | 0.915 | 0.0136 | 0.178 |
| *CSC001* | 17 | 11/1 | 7.961 | 0.588 | 0.875 | 0.0005* | 0.355 | *CSC001* | 20 | 8/0 | 6.29 | 0.150 | 0.828 | **0.0000*** | 0.827 |
| *CSA035* | 18 | 20/1 | 11.222 | 0.722 | 0.940 | 0.0010* | 0.258 | *CSA035* | 22 | 25/1 | 11.594 | 0.773 | 0.948 | **0.0000*** | 0.208 |
| *CSC004* | 18 | 20/1 | 11.408 | 0.611 | 0.943 | **0.0000*** | 0.377 | *CSC004* | 21 | 16/1 | 9.569 | 0.524 | 0.915 | **0.0000*** | 0.447 |
| Pen23472 | 13 | 4/0 | 4.486 | 1.000 | 0.595 | 0.0032* | -0.024 | Pen23472 | 22 | 5/0 | 4.743 | 0.955 | 0.772 | 0.0996 | -0.215 |

Table SM2 (continued)

| Chesapeake Bay | | | | | | | | Slidell, Louisiana | | | | | | | |
| --- | --- | --- | --- | --- | --- | --- | --- | --- | --- | --- | --- | --- | --- | --- | --- |
| Primer | N | N_A_/N_P_ | AR | H_O_ | H_E_ | P_HW_ | F_IS_ | Primer | N | N_A_/N_P_ | AR | H_O_ | H_E_ | P_HW_ | F_IS_ |
| Tet6290 | 25 | 4/0 | 2.504 | 0.360 | 0.334 | 0.3868 | -0.059 | Tet6290 | 10 | 2/0 | 1.921 | 0.200 | 0.180 | 1.0000 | -0.059 |
| Tet1329 | 25 | 4/0 | 2.878 | 0.440 | 0.416 | 0.1311 | -0.037 | Tet1329 | 11 | 4/1 | 3.271 | 0.364 | 0.479 | 0.2575 | 0.286 |
| CSA121 | 25 | 5/1 | 2.962 | 0.360 | 0.370 | 0.7551 | 0.046 | CSA121 | 11 | 3/0 | 2.6 | 0.182 | 0.310 | 0.2771 | 0.452 |
| Tet603 | 24 | 9/1 | 5.366 | 0.833 | 0.725 | 0.5464 | -0.129 | Tet603 | 11 | 4/0 | 3.506 | 0.545 | 0.492 | 0.3065 | -0.062 |
| Di680 | 18 | 13/0 | 8.782 | 0.778 | 0.895 | 0.0535 | 0.159 | Di680 | 11 | 12/0 | 8.97 | 0.727 | 0.851 | 0.0176 | 0.192 |
| CSC094 | 25 | 6/0 | 4.223 | 0.560 | 0.657 | 0.1815 | 0.167 | CSC094 | 11 | 4/0 | 3.954 | 0.545 | 0.707 | 0.2105 | 0.273 |
| CSA073 | 22 | 22/2 | 10.617 | 0.909 | 0.932 | 0.0751 | 0.048 | CSA073 | 10 | 16/1 | 11.925 | 1.000 | 0.925 | 1.0000 | -0.029 |
| Tri24376 | 25 | 6/0 | 4.428 | 0.720 | 0.691 | 0.4682 | -0.021 | Tri24376 | 11 | 7/0 | 5.776 | 0.818 | 0.769 | 0.7260 | -0.017 |
| *Tet1886* | 20 | 7/0 | 4.984 | 0.400 | 0.740 | 0.0030* | 0.544 | *Tet1886* | 7 | 5/0 | 5 | 0.286 | 0.786 | 0.0176 | 0.68 |
| *Tet1057* | 24 | 7/2 | 4.672 | 0.417 | 0.708 | 0.0019* | 0.429 | Tet1057 | 11 | 4/0 | 3.236 | 0.364 | 0.380 | 0.4374 | 0.091 |
| *Pen9028* | 25 | 8/0 | 6.191 | 0.520 | 0.810 | 0.0012* | 0.376 | *Pen9028* | 11 | 7/0 | 5.984 | 0.455 | 0.760 | 0.0118 | 0.441 |
| *CSC007* | 25 | 18/0 | 9.625 | 0.680 | 0.915 | 0.0004* | 0.276 | CSC007 | 11 | 12/0 | 9.903 | 0.818 | 0.909 | 0.1932 | 0.147 |
| *CSC001* | 25 | 11/0 | 8.12 | 0.280 | 0.888 | **0.0000*** | 0.695 | *CSC001* | 9 | 7/0 | 6.472 | 0.333 | 0.815 | 0.0012* | 0.628 |
| *CSA035* | 22 | 22/1 | 11.017 | 0.727 | 0.940 | 0.0004* | 0.248 | *CSA035* | 10 | 11/0 | 8.884 | 0.600 | 0.855 | 0.0003* | 0.345 |
| *CSC004* | 24 | 23/1 | 11.006 | 0.792 | 0.940 | **0.0000*** | 0.179 | *CSC004* | 11 | 14/0 | 10.475 | 0.636 | 0.909 | 0.0058 | 0.343 |
| Pen23472 | 25 | 7/0 | 5.579 | 0.760 | 0.818 | 0.0005* | 0.091 | Pen23472 | 11 | 5/0 | 4.477 | 0.636 | 0.669 | 0.1683 | 0.097 |
| Lagoa dos Patos, Brazil | | | | | | | | Tramandaí, Brazil | | | | | | | |
| Primer | N | N_A_/N_P_ | AR | H_O_ | H_E_ | P_HW_ | F_IS_ | Primer | N | N_A_/N_P_ | AR | H_O_ | H_E_ | P_HW_ | F_IS_ |
| CSA121 | 57 | 3/1 | 1.730 | 0.070 | 0.101 | 0.0037* | 0.3139 | CSA121 | 33 | 2/0 | 2.534 | 0.121 | 0.114 | 1.000 | -0.0492 |
| CSC094 | 57 | 5/0 | 2.204 | 0.263 | 0.240 | **0.0012*** | -0.086 | CSC094 | 25 | 3/0 | 2.684 | 0.120 | 0.183 | 0.1977 | 0.3628 |
| CSA073 | 58 | 26/0 | 5.689 | 0.828 | 0.795 | 0.0371 | -0.0317 | CSA073 | 34 | 12/1 | 12.563 | 0.794 | 0.710 | **0.0000*** | -0.1041 |
| CSC007 | 55 | 26/1 | 11.973 | 0.855 | 0.924 | **0.0006*** | 0.0844 | CSC007 | 28 | 23/0 | 12.359 | 0.929 | 0.926 | 0.0556 | 0.0154 |
| CSC001 | 56 | 13/2 | 5.139 | 0.250 | 0.583 | **0.0000*** | 0.5775 | CSC001 | 24 | 7/1 | 5.919 | 0.208 | 0.510 | **0.0002*** | 0.6055 |
| CSA035 | 54 | 20/1 | 5.220 | 0.685 | 0.724 | 0.0628 | 0.0633 | CSA035 | 33 | 11/0 | 6.340 | 0.485 | 0.517 | 0.2868 | 0.0775 |
| CSC004 | 54 | 14/2 | 5.501 | 0.870 | 0.774 | 1.0000 | -0.1158 | CSC004 | 32 | 10/1 | 5.234 | 0.688 | 0.734 | 0.1091 | 0.0796 |
| Itajaí, Brazil | | | | | | | | Laguna, Brazil | | | | | | | |
| Primer | N | N_A_/N_P_ | AR | H_O_ | H_E_ | P_HW_ | F_IS_ | Primer | N | N_A_/N_P_ | AR | H_O_ | H_E_ | P_HW_ | F_IS_ |
| CSA121 | 43 | 3/0 | 1.503 | 0.140 | 0.250 | **0.0012*** | 0.4510 | CSA121 | 67 | 3/0 | 2.680 | 0.060 | 0.058 | 1.000 | -0.0154 |
| CSC094 | 45 | 4/0 | 2.680 | 0.333 | 0.400 | 0.0695 | 0.1776 | CSC094 | 67 | 5/0 | 5.049 | 0.388 | 0.346 | 0.9077 | -0.1132 |
| CSA073 | 53 | 21/6 | 8.647 | 0.962 | 0.773 | **0.0013*** | -0.2352 | CSA073 | 66 | 28/5 | 11.347 | 0.758 | 0.776 | 0.0019* | 0.0317 |
| CSC007 | 40 | 26/1 | 12.866 | 0.950 | 0.938 | 0.0189 | -0.0003 | CSC007 | 67 | 33/4 | 12.579 | 0.925 | 0.949 | **0.0001*** | 0.0322 |
| CSC001 | 40 | 9/1 | 6.194 | 0.450 | 0.673 | **0.0000*** | 0.3430 | CSC001 | 67 | 16/2 | 9.714 | 0.358 | 0.600 | **0.0000*** | 0.409 |
| CSA035 | 52 | 17/0 | 6.636 | 0.635 | 0.604 | 0.7543 | -0.0402 | CSA035 | 65 | 22/3 | 10.707 | 0.646 | 0.669 | 0.258 | 0.0419 |
| CSC004 | 43 | 10/3 | 6.135 | 0.767 | 0.729 | **0.0012*** | -0.0417 | CSC004 | 66 | 19/2 | 10.427 | 0.773 | 0.747 | 0.0475 | -0.0266 |

Table SM3. Genetic diversity, inbreeding coefficient, and percentage of missing data, per locus in US and Brazilian localities. Rows marked by an asterisk comprise the “US-seven-loci-dataset”

| *Locus* | *Country* | *N_A_* | *H_O_* | *H_S_* | *H_T_* | *H_T_’* | *G_IS_* | *% Missing* | *NA freq* |
| --- | --- | --- | --- | --- | --- | --- | --- | --- | --- |
| *Tet6290 | US | 6 | 0.288 | 0.272 | 0.273 | 0.273 | -0.057 | 2.7 | 0.0000 |
| *Tet1329 | US | 7 | 0.548 | 0.504 | 0.519 | 0.521 | -0.089 | 0.0 | 0.0000 |
| *CSA121 | US | 7 | 0.320 | 0.338 | 0.335 | 0.335 | 0.055 | 2.2 | 0.0049 |
| *Tet603 | US | 12 | 0.718 | 0.676 | 0.684 | 0.685 | -0.062 | 2.7 | 0.0000 |
| *Di680 | US | 21 | 0.824 | 0.918 | 0.924 | 0.925 | 0.102 | 5.9 | 0.0501 |
| *CSC094 | US | 7 | 0.602 | 0.673 | 0.670 | 0.669 | 0.106 | 2.2 | 0.0373 |
| *CSA073 | US | 47 | 0.870 | 0.953 | 0.954 | 0.955 | 0.087 | 8.1 | 0.041 |
| Tri24376 | US | 11 | 0.506 | 0.721 | 0.717 | 0.716 | 0.298 | 3.2 | 0.1326 |
| Tet1886 | US | 10 | 0.386 | 0.747 | 0.756 | 0.757 | 0.483 | 11.3 | 0.2205 |
| Tet1057 | US | 8 | 0.363 | 0.637 | 0.638 | 0.638 | 0.430 | 3.8 | 0.2059 |
| Pen9028 | US | 11 | 0.556 | 0.762 | 0.759 | 0.758 | 0.270 | 0.5 | 0.1192 |
| CSC007 | US | 36 | 0.804 | 0.945 | 0.948 | 0.949 | 0.149 | 1.1 | 0.0774 |
| CSC001 | US | 17 | 0.437 | 0.906 | 0.903 | 0.903 | 0.518 | 8.1 | 0.258 |
| CSA035 | US | 47 | 0.700 | 0.961 | 0.963 | 0.963 | 0.271 | 3.2 | 0.1274 |
| CSC004 | US | 36 | 0.662 | 0.958 | 0.960 | 0.960 | 0.309 | 2.7 | 0.1452 |
| Pen23472 | US | 7 | 0.791 | 0.761 | 0.793 | 0.796 | -0.040 | 2.2 | 0.0000 |
| **Average**  **US 16 loci** | **US** | **18.12** | **0.59** | **0.73** | **0.74** | **0.74** | **0.18** | **3.74** | **0.0887** |
|  |  |  |  |  |  |  |  |  |  |
| CSA121 | Brazil | 3 | 0.074 | 0.082 | 0.082 | 0.082 | 0.096 | na |  |
| CSC094 | Brazil | 6 | 0.276 | 0.296 | 0.301 | 0.303 | 0.068 | na |  |
| CSA073 | Brazil | 43 | 0.835 | 0.770 | 0.774 | 0.775 | 0.084 | na |  |
| CSC007 | Brazil | 42 | 0.915 | 0.934 | 0.936 | 0.937 | 0.020 | na |  |
| CSC001 | Brazil | 24 | 0.317 | 0.582 | 0.588 | 0.591 | 0.0456 | na |  |
| CSA035 | Brazil | 33 | 0.613 | 0.635 | 0.639 | 0.640 | 0.036 | na |  |
| CSC004 | Brazil | 31 | 0.775 | 0.747 | 0.747 | 0.747 | 0.037 | na |  |
| **Average Brazil 7 loci** | **Brazil** | **26** | **0.54** | **0.58** | **0.58** | **0.58** | **0.055** |  |  |

*N_A_* = Mean number of alleles; *H_O_* = mean observed heterozygosity within populations; *H_S_* = mean expected heterozygosity within populations; *H_T_* = total heterozygosity, *H_T_’* = corrected total heterozygosity; *G_IS_* = inbreeding coefficient; NA freq = frequency of null alleles pooling data from all populations (underlined numbers indicate that frequency of null alleles was not significant at p < 0.05).

Table SM4. Global Jost’s differentiation (*D_ST_*), corrected Jost’s differentiation (*D_ST_’*), fixation index (*G_ST_*), and Nei’s corrected fixation index (*G_ST_’*) per locus in the US and Brazil

| *Locus* | *Country* | *D_ST_* | *D_ST_’* | *G_ST_* | *G_ST_’* |
| --- | --- | --- | --- | --- | --- |
| Tet6290 | US | 0.001 | 0.001 | 0.002 | 0.002 |
| Tet1329 | US | 0.015 | 0.017 | 0.030 | 0.033 |
| CSA121 | US | -0.003 | -0.003 | -0.008 | -0.009 |
| Tet603 | US | 0.008 | 0.009 | 0.012 | 0.013 |
| Di680 | US | 0.007 | 0.007 | 0.007 | 0.008 |
| CSC094 | US | -0.004 | -0.004 | -0.006 | -0.006 |
| CSA073 | US | 0.002 | 0.002 | 0.002 | 0.002 |
| Tri24376 | US | -0.004 | -0.004 | -0.005 | -0.006 |
| Tet1886 | US | 0.009 | 0.010 | 0.012 | 0.014 |
| Tet1057 | US | 0.001 | 0.001 | 0.001 | 0.001 |
| Pen9028 | US | -0.003 | -0.003 | -0.004 | -0.004 |
| CSC007 | US | 0.003 | 0.003 | 0.003 | 0.004 |
| CSC001 | US | -0.002 | -0.003 | -0.003 | -0.003 |
| CSA035 | US | 0.002 | 0.002 | 0.002 | 0.002 |
| CSC004 | US | 0.003 | 0.003 | 0.003 | 0.003 |
| **Average US 15 loci** | **US** | **0.002** | **0.002** | **0.003** | **0.004** |
| Pen23472 | US | 0.032 | 0.036 | 0.040 | 0.045 |
|  |  |  |  |  |  |
| CSA121 | Brazil | 0.000 | 0.000 | 0.004 | 0.005 |
| CSC094 | Brazil | 0.005 | 0.007 | 0.017 | 0.022 |
| CSA073 | Brazil | 0.003 | 0.004 | 0.004 | 0.006 |
| CSC007 | Brazil | 0.003 | 0.004 | 0.003 | 0.004 |
| CSC001 | Brazil | 0.006 | 0.009 | 0.011 | 0.014 |
| CSA035 | Brazil | 0.003 | 0.005 | 0.005 | 0.007 |
| CSC004 | Brazil | 0.000 | 0.000 | 0.000 | 0.001 |
| **Average Brazil** | **Brazil** | **0.003** | **0.004** | **0.006** | **0.008** |

**Table SM5**. Pairwise *F_ST_* values between US localities calculated using the private alleles method for the US-seven-loci dataset (above the diagonal) and for all 15 neutral loci (below the diagonal). No value was significant after Bonferroni correction (p < 0.0011) nor at a FDR ≤ 0.05.

|  | SERC | CEK | APA | DIB | SLI | AVI | GAL | POL | ROC | LLM |
| --- | --- | --- | --- | --- | --- | --- | --- | --- | --- | --- |
| SERC |  | -0.00571 | -0.01664 | -0.01334 | -0.00113 | -0.00647 | -0.00424 | 0.02364 | -0.01148 | -0.0013 |
| CEK | 0.00391 |  | 0.00197 | -0.00383 | 0.01224 | -0.01291 | 0.01954 | 0.00708 | -0.00341 | 0.00371 |
| APA | 0.00099 | 0.01962 |  | -0.00853 | -0.00476 | 0.00387 | 0.01396 | 0.01769 | -0.00470 | -0.01151 |
| DIB | -0.00294 | -0.00024 | 0.00050 |  | 0.00046 | 0.00454 | 0.01556 | 0.01871 | -0.00782 | -0.00299 |
| SLI | 0.00958 | 0.01989 | 0.00289 | 0.01072 |  | 0.00008 | 0.00465 | 0.01261 | -0.00944 | -0.00685 |
| AVI | -0.00152 | 0.00554 | 0.00290 | 0.00186 | 0.00840 |  | 0.00150 | 0.00857 | -0.00463 | -0.00638 |
| GAL | 0.00178 | 0.00970 | 0.01222 | 0.01104 | 0.00071 | 0.00522 |  | 0.04307 | 0.00282 | 0.01056 |
| POL | 0.01476 | 0.00529 | 0.01084 | 0.00755 | 0.01386 | 0.00485 | 0.02012 |  | 0.01076 | 0.01378 |
| ROC | -0.00035 | 0.00566 | 0.00467 | 0.00024 | 0.00536 | 0.00495 | 0.00141 | 0.00903 |  | -0.01099 |
| LLM | 0.00010 | 0.00707 | 0.00015 | -0.00258 | 0.00612 | 0.00038 | -0.00048 | 0.00596 | -0.00481 |  |

**Table SM6**. Pairwise F_ST_ values between US localities using locus *Pen23472*. Values with * are significant after Bonferroni correction (*p* < 0.001); values in bold are significant at a FDR ≤ 0.05.

|  | APA | AVI | CEK | DIB | GAL | LLM | POL | ROC | SERC | SLI |
| --- | --- | --- | --- | --- | --- | --- | --- | --- | --- | --- |
| APA |  |  |  |  |  |  |  |  |  |  |
| AVI | **0.16746*** |  |  |  |  |  |  |  |  |  |
| CEK | -0.02061 | **0.10457** |  |  |  |  |  |  |  |  |
| DIB | 0.01845 | **0.19297*** | 0.02296 |  |  |  |  |  |  |  |
| GAL | 0.05356 | **0.11006** | 0.04009 | 0.02782 |  |  |  |  |  |  |
| LLM | **0.09252** | 0.06505 | 0.06003 | **0.08927** | -0.01400 |  |  |  |  |  |
| POL | 0.04456 | 0.04938 | 0.00733 | 0.07435 | 0.01086 | 0.00075 |  |  |  |  |
| ROC | **0.14885*** | **0.09853** | **0.09011** | **0.18217*** | **0.11240** | **0.07067** | 0.01762 |  |  |  |
| SERC | 0.03668 | 0.06723 | 0.00880 | 0.03952 | -0.01430 | 0.00586 | -0.01310 | 0.05767 |  |  |
| SLI | 0.04497 | **0.17583** | 0.05508 | 0.00858 | -0.02223 | 0.03797 | 0.06542 | **0.20105*** | 0.02221 |  |

Table SM7. AMOVA results for different groupings within the US and for US vs. Brazil based on the 15-loci dataset.

GOM = Gulf of Mexico; CB = Chesapeake Bay; CI = Confidence interval.

| Group | Source of Variation | % Variation | F-stat | F-value | CI 2.5% | CI 97.5% | P-value |
| --- | --- | --- | --- | --- | --- | --- | --- |
| US (15 loci) | Within individuals | 78.9 | F_it | 0.211 | 0.122 | 0.299 | < 0.001 |
|  | Among individuals within localities | 20.8 | F_is | 0.208 | 0.119 | 0.298 | < 0.001 |
|  | Among populations within the U.S. | 0.3 | F_st | 0.003 | -0.001 | 0.007 | 0.999 |
| GOM (15 loci) | Within individuals | 79.1 | F_it | 0.209 | 0.119 | 0.298 | <0.001 |
|  | Among individuals within localities | 20.6 | F_is | 0.206 | 0.115 | 0.295 | <0.001 |
|  | Among populations within the GOM | 0.3 | F_st | 0.003 | -0.001 | 0.008 | 0.999 |
| GOM vs. CB (15 loci) | Within individuals | 78.9 | F_it | 0.210 | 0.122 | 0.299 | <0.001 |
|  | Among individuals within populations | 20.8 | F_is | 0.208 | 0.119 | 0.299 | <0.001 |
|  | Among populations within each group | 0.3 | F_sc | 0.003 | -0.001 | 0.007 | 0.069 |
|  | Between GOM *vs*. CB | -0.1 | F_ct | -0.003 | -0.006 | 0.004 | 0.532 |
| West vs. East GOM (15 loci) | Within individuals | 79.1 | F_it | 0.209 | 0.119 | 0.297 | <0.001 |
|  | Among individuals within localities | 20.6 | F_is | 0.207 | 0.116 | 0.295 | <0.001 |
|  | Among populations within each group | 0.31 | F_sc | 0.003 | -0.002 | 0.009 | 0.076 |
|  | Between West *vs*. East GOM | -0.01 | F_ct | -0.0001 | -0.002 | 0.002 | 0.449 |
| US vs. BRAZIL (7 common loci) | Within individuals | 44.4 | R_it | 0.556 | 0.454 | 0.609 | -- |
|  | Among individuals within localities | 40.5 | R_is | 0.477 | 0.339 | 0.575 | 0.001 |
|  | Among populations within each group | 0.5 | R_sc | 0.006 | 0.001 | 0.011 | 0.552 |
|  | Between US & BRAZIL | 14.5 | R_ct | 0.145 | 0.049 | 0.250 | 0.001 |

Table SM8. Pairwise *F_ST_* values calculated with FreeNA for US and Brazilian localities with (below diagonal) and without (above diagonal) correction for null alleles using the seven common loci between the two studies

|  | APA | AVI | CEK | DIB | GAL | LLM | POL | ROC | SERC | SLI | LPA | TRA | ITA | LAG |
| --- | --- | --- | --- | --- | --- | --- | --- | --- | --- | --- | --- | --- | --- | --- |
| APA | -- | 0.00627 | 0.005372 | -0.004576 | 0.004548 | 0.002501 | -0.00232 | 0.000126 | -0.002922 | 0.000055 | **0.176747** | **0.205076** | **0.156798** | **0.169967** |
| AVI | -0.002431 | -- | 0.00627 | 0.00491 | -0.0008 | 0.00825 | -0.0023 | 0.00436 | -0.0056 | 0.00409 | **0.14783** | **0.1802** | **0.13517** | **0.14392** |
| CEK | 0.006781 | 0.01359 | -- | -0.007979 | -0.005523 | -0.003038 | -0.002659 | 0.002422 | -0.006804 | -0.000344 | **0.154109** | **0.183627** | **0.133362** | **0.150437** |
| DIB | -0.004524 | 0.00386 | -0.004838 | -- | 0.002521 | -0.003227 | 0.000314 | -0.000955 | -0.004624 | 0.001845 | **0.160973** | **0.181741** | **0.140482** | **0.156023** |
| GAL | 0.005633 | 0.0045 | 0.000929 | 0.002062 | -- | -0.001133 | -0.003319 | -0.000525 | -0.005537 | -0.001049 | **0.12543** | **0.160641** | **0.115728** | **0.127553** |
| LLM | 0.002045 | 0.00903 | 0.000625 | -0.001113 | 0.001399 | -- | 0.002185 | -0.003865 | -0.001175 | -0.008101 | **0.173807** | **0.197551** | **0.152749** | **0.172057** |
| POL | -0.001718 | 0.00018 | -0.002083 | -0.002378 | -0.001075 | 0.003591 | -- | 0.003576 | -0.004726 | -0.00502 | **0.148855** | **0.175704** | **0.131031** | **0.145885** |
| ROC | -0.001477 | 0.00364 | 0.005199 | -0.002668 | 0.000925 | -0.002965 | 0.003791 | -- | -0.000884 | -0.006786 | **0.168551** | **0.194403** | **0.150065** | **0.166586** |
| SERC | -0.001422 | -0.002 | 0.000087 | -0.003448 | -0.000788 | 0.000072 | -0.004935 | -0.001529 | -- | 0.000072 | **0.15215** | **0.173439** | **0.133221** | **0.151206** |
| SLI | -0.002579 | 0.00313 | 0.000439 | 0.000122 | -0.002642 | -0.00699 | -0.004422 | -0.00526 | 0.000666 | -- | **0.182802** | **0.214656** | **0.157577** | **0.178764** |
| **LPA** | **0.17222** | **0.14705** | **0.157462** | **0.153346** | **0.124529** | **0.175419** | **0.144867** | **0.161135** | **0.151553** | **0.170796** | -- | 0.004079 | 0.010141 | 0.004201 |
| **TRA** | **0.19275** | **0.17506** | **0.181193** | **0.168545** | **0.152116** | **0.19169** | **0.166634** | **0.182325** | **0.169853** | **0.196081** | 0.005016 | -- | 0.007433 | 0.008077 |
| **ITA** | **0.1489** | **0.13204** | **0.135273** | **0.13384** | **0.112227** | **0.14959** | **0.127915** | **0.143427** | **0.13188** | **0.145** | 0.012891 | 0.008491 | -- | 0.005149 |
| **LAG** | **0.166981** | **0.14447** | **0.154465** | **0.150426** | **0.126357** | **0.17392** | **0.143791** | **0.160462** | **0.151362** | **0.168259** | 0.00349 | 0.007803 | 0.00846 | -- |

Bolded values indicate US vs. Brazil comparisons. All US vs. Brazil pairwise comparisons without correction (above diagonal) were significant at *p* = 0.05

Table SM9. *N_e_* estimations for blue crabs in the US, GOM and Brazil

| Region | No. of loci | Method | Lowest Allele Frequency Used | N_E_ | 95% CI for Ne |
| --- | --- | --- | --- | --- | --- |
| US | 15 | Heterozygote Excess Method | 0.05 | Infinite | Infinite |
|  |  | Heterozygote Excess Method | 0.02 | Infinite | Infinite |
|  |  | Heterozygote Excess Method | 0.01 | Infinite | Infinite |
|  |  | LD Method | 0.05 | Infinite | 1024.8 – Infinite |
|  |  | LD Method | 0.02 | 5140.5 | 1351.1 – Infinite |
|  |  | LD Method | 0.01 | 2603.9 | 1250.0 – Infinite |
| GOM | 15 | Heterozygote Excess Method | 0.05 | Infinite | Infinite |
|  |  | Heterozygote Excess Method | 0.02 | Infinite | Infinite |
|  |  | Heterozygote Excess Method | 0.01 | Infinite | Infinite |
|  |  | LD Method | 0.05 | Infinite | 1089.5 – Infinite |
|  |  | LD Method | 0.02 | 4550.3 | 1201.8 – Infinite |
|  |  | LD Method | 0.01 | 2160.8 | 1055.6 – Infinite |
| Brazil | 7 | Heterozygote Excess Method | 0.05 | Infinite | Infinite |
|  |  | Heterozygote Excess Method | 0.020 | Infinite | Infinite |
|  |  | Heterozygote Excess Method | 0.010 | Infinite | Infinite |
|  |  | LD Method | 0.05 | -275.0 | -1340.4 – Infinite |
|  |  | LD Method | 0.020 | -1473.3 | 944.5 – Infinite |
|  |  | LD Method | 0.010 | -5027.4 | 1359.4 – Infinite |

Table SM10. BOTTLENECK results using the 15 putatively neutral loci for the 10 US localities

| Locality | Wilcoxon Test | | |  | A.D.T. |  |
| --- | --- | --- | --- | --- | --- | --- |
|  | IAM | TPM | SMM |  | L-shape | |
| APA | 0.31934 | 0.98492 | 0.99582 |  | L-shape | |
| AVI | 0.04730 | 0.89612 | 0.96814 |  | L-shape | |
| CEK | 0.13843 | 0.66061 | 0.80530 |  | L-shape | |
| DIB | 0.06769 | 0.99377 | 0.99934 |  | L-shape | |
| GAL | 0.06769 | 0.95270 | 0.92807 |  | L-shape | |
| LLM | 0.17957 | 0.99097 | 0.99832 |  | L-shape | |
| POL | **0.00050** | 0.28070 | 0.38077 |  | L-shape | |
| ROC | **0.00005** | 0.66061 | 0.82043 |  | L-shape | |
| SERC | 0.04730 | 0.91559 | 0.96350 |  | L-shape | |
| SLI | 0.44519 | 0.947708 | 0.958374 |  | L-shape | |

Bolded values indicate significance after Bonferroni correction( *p* < 0.005) and at a FDR ≤ 0.05.

L-shape distribution does not suggest a recent bottleneck.

**Table SM11**. Point estimates and 95% confidence intervals (in squared brackets) of the demographic parameters estimated with MIGRAINE using the US-seven-loci dataset (GSM model) and the 15 neutral loci (GSM and SMM/GSM models)

|  |  |  |  | Parameter/CI |  |  |  |
| --- | --- | --- | --- | --- | --- | --- | --- |
| Dataset | Model | pGSM | θ_cur_ | Dg/2N | θ_anc_ | Nratio | Dg*μ |
| 7 loci | GSM | 0.562  [0.46 - 0.651] | 14.01  [9.482 - 19.78] | 0.0643  [0.0176 - 0.635] | 1.365  [NA - 4.378] | 10.27  [2.983 - 19765] | 0.901  [0.313 - 6.832] |
| 15 loci | GSM | 0.51  [0.424 - 0.582] | 24.48  [18.52 - 34.39] | 0.036  [0.0132 - 0.079] | 4.61  [1.528 - 9.37] | 5.311  [2.483 - 15.44] | 0.883  [0.392 - 1.649] |
| 15 loci | SMM/GSM | 0.737  [0.679 - 0.786] | 35.3  [24.28 - 65.63] | 0.0154  [0.00282 - 0.0513] | 11.57  [7.596 - 16.8] | 3.05  [1.701 - 5.458] | 0.544  [0.161 - 1.382] |
|  |  |  |  |  |  |  |  |

Notes:

Converted parameters using µ=0.0005 for 15 loci with the GSM model:

N: 12,240 (9,260 – 17, 160) diploid individuals

Dg from Dg/2N: 1,762 (646 – 3,867) generations

Nanc: 2,305 (764 – 4,685) diploid individuals

Dg from Dgμ: 1,766 (784 – 3,298) generations
