# Supplementary material for: Population genetics and historical demographic inferences of the blue crab Callinectes sapidus in the US based on microsatellites
Source: PeerJ. 2019 Oct 14;7:e7780. doi: 10.7717/peerj.7780 (PMC6796965; doi:10.7717/peerj.7780)
Supplement: Supplemental Information 2 [file peerj-07-7780-s002.pdf]

a) b)

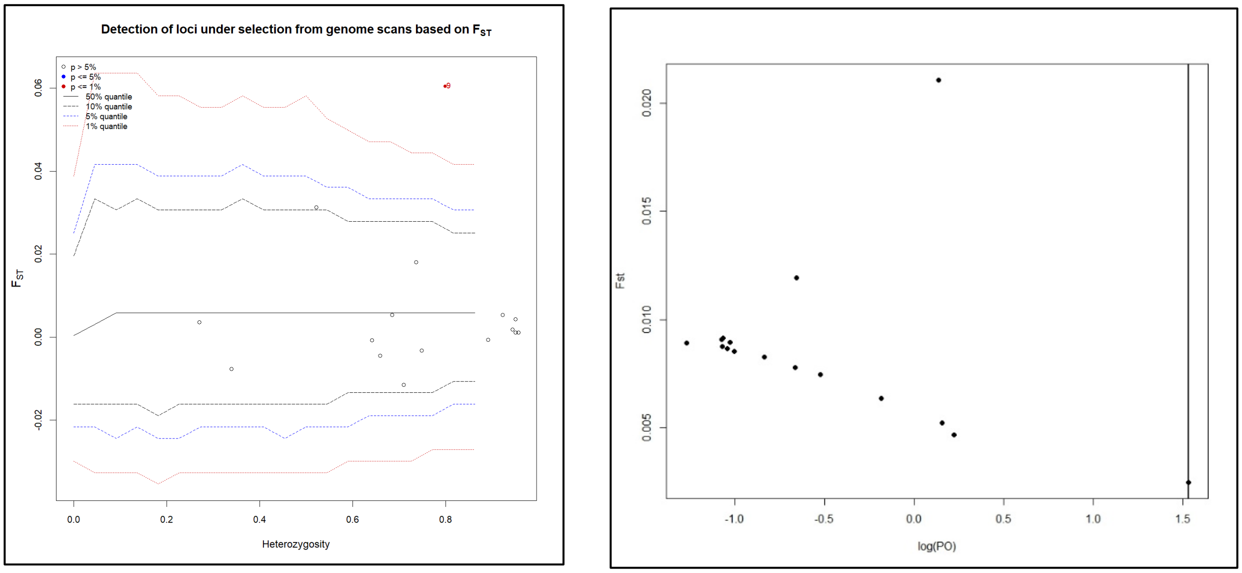

Figure SM1. Tests for loci under putative selection. a) Test of Beaumont and Nichols (1996) implemented in ARLEQUIN v.3.5.2.1; b) test of Beaumont and Balding (2004) implemented in Bayescan v.2.1. Both tests detected *Pen23472* as the only outlier locus.

a)

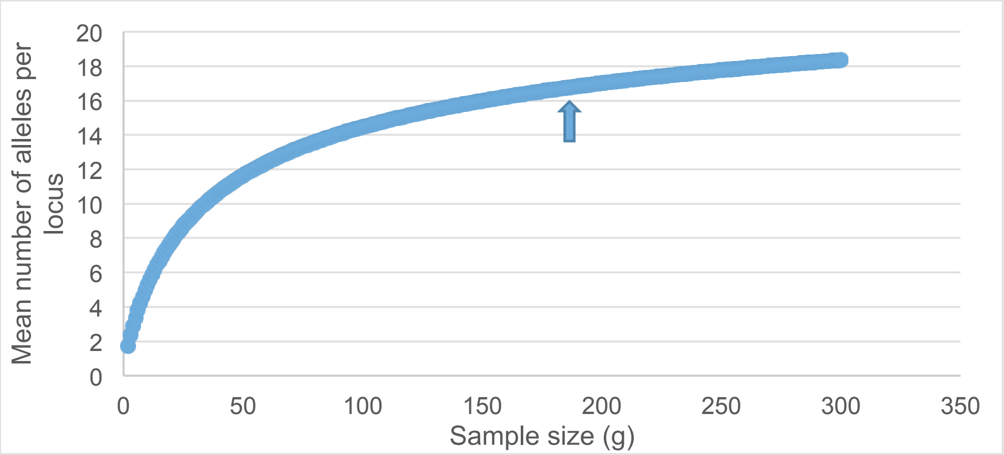

b)

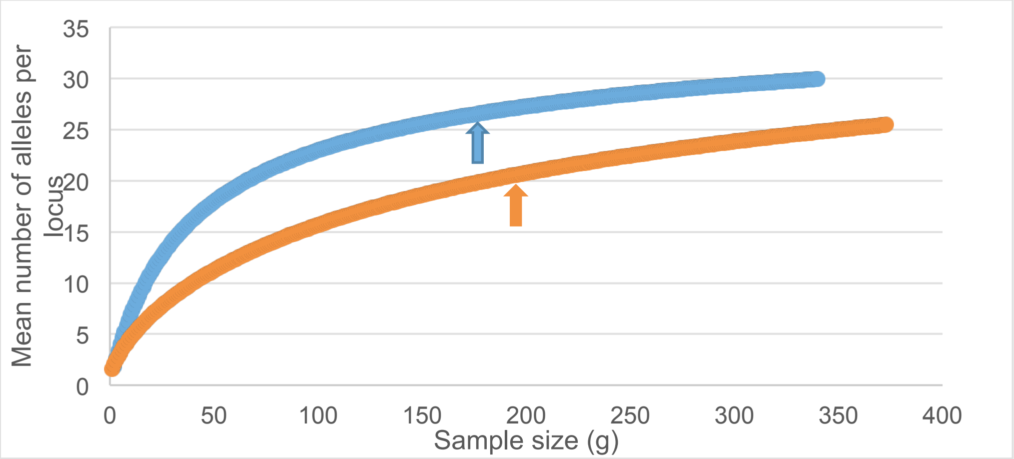

Figure SM2. ADZE rarefaction analyses. a) for the US using all 16 loci; and b) for the US (blue line) and Brazil (orange line) using the seven loci in common for both studies. The arrows indicate where the used sample sizes fall along the curve.

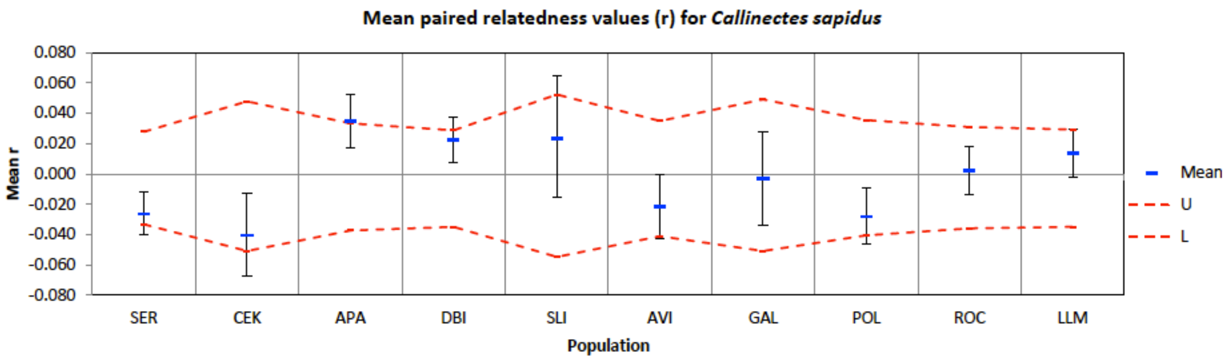

Figure SM3. Mean paired relatedness for the blue crab in the 10 US localities.

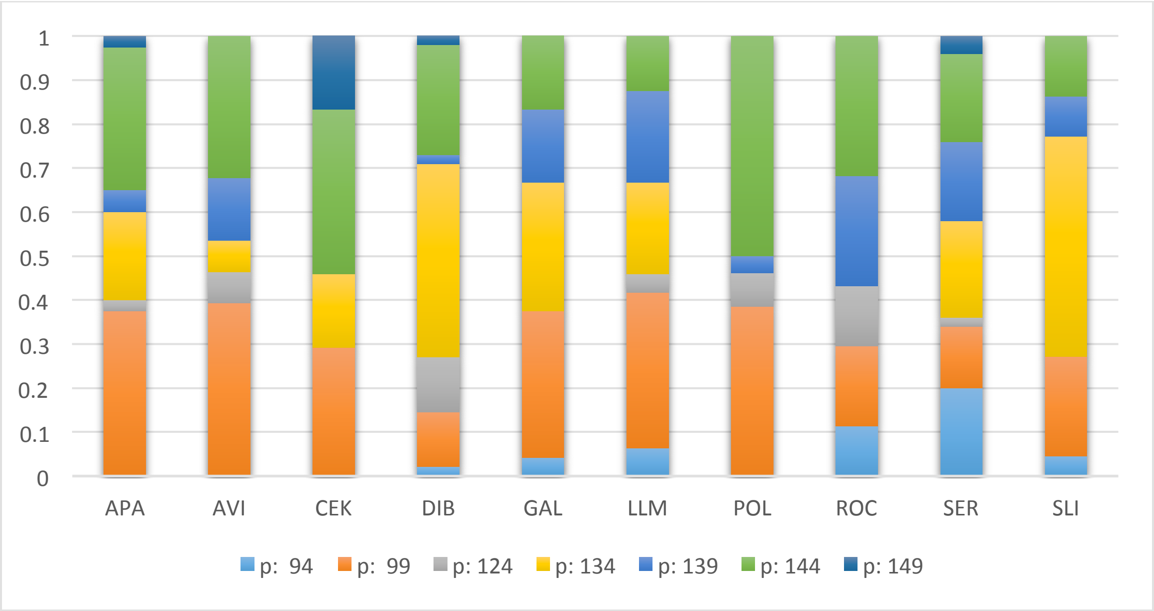

Figure SM4. Frequencies of the seven alleles at the *Pen23472* locus at each of the ten US localities.

a.

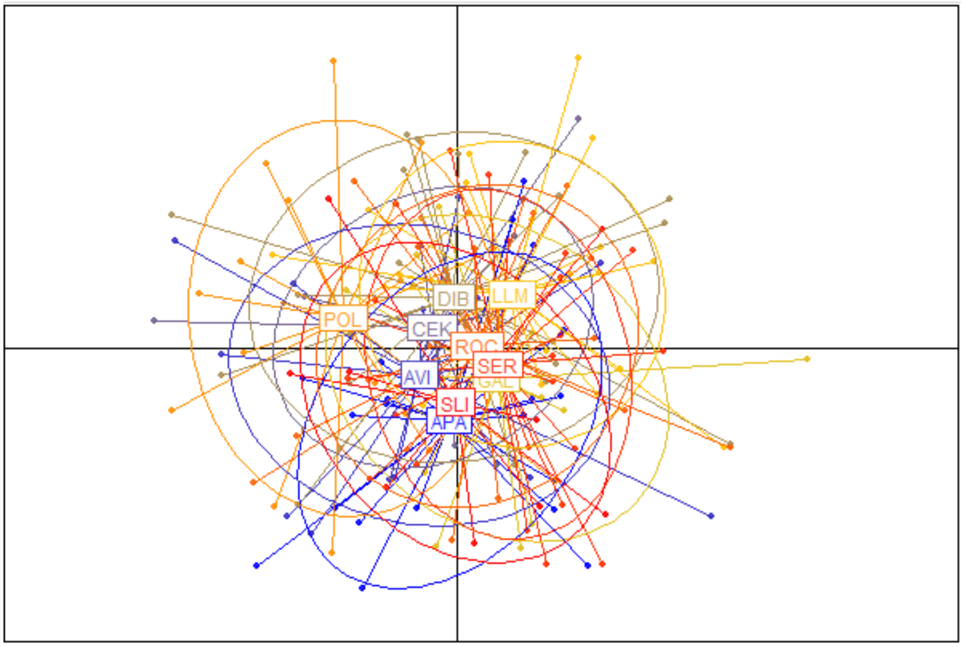

b.

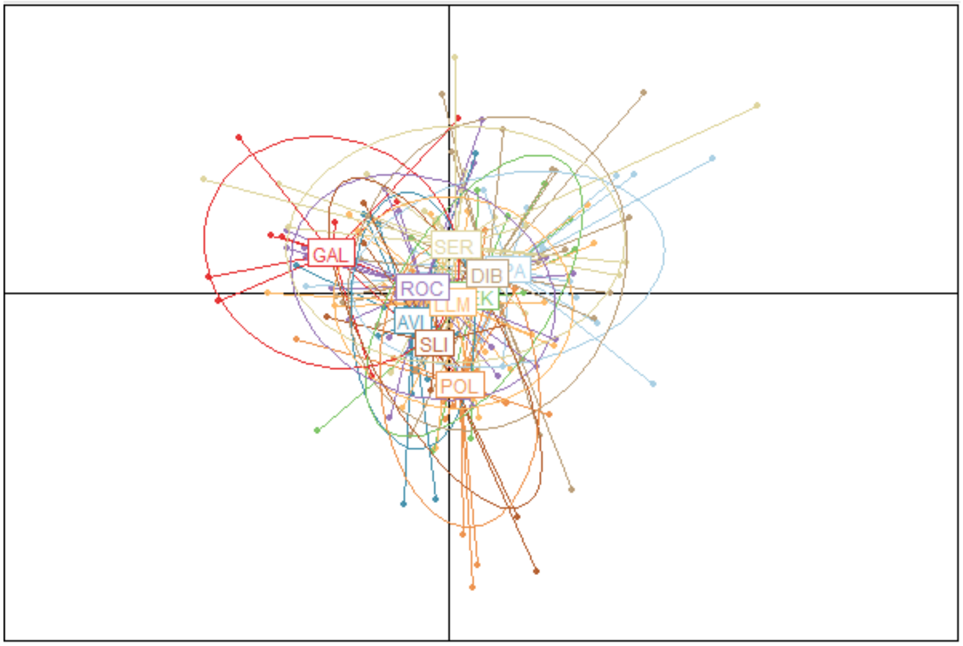

Figure SM5. DAPC analyses. a) Using all 15 putatively neutral loci: b) using the US-seven-loci-dataset

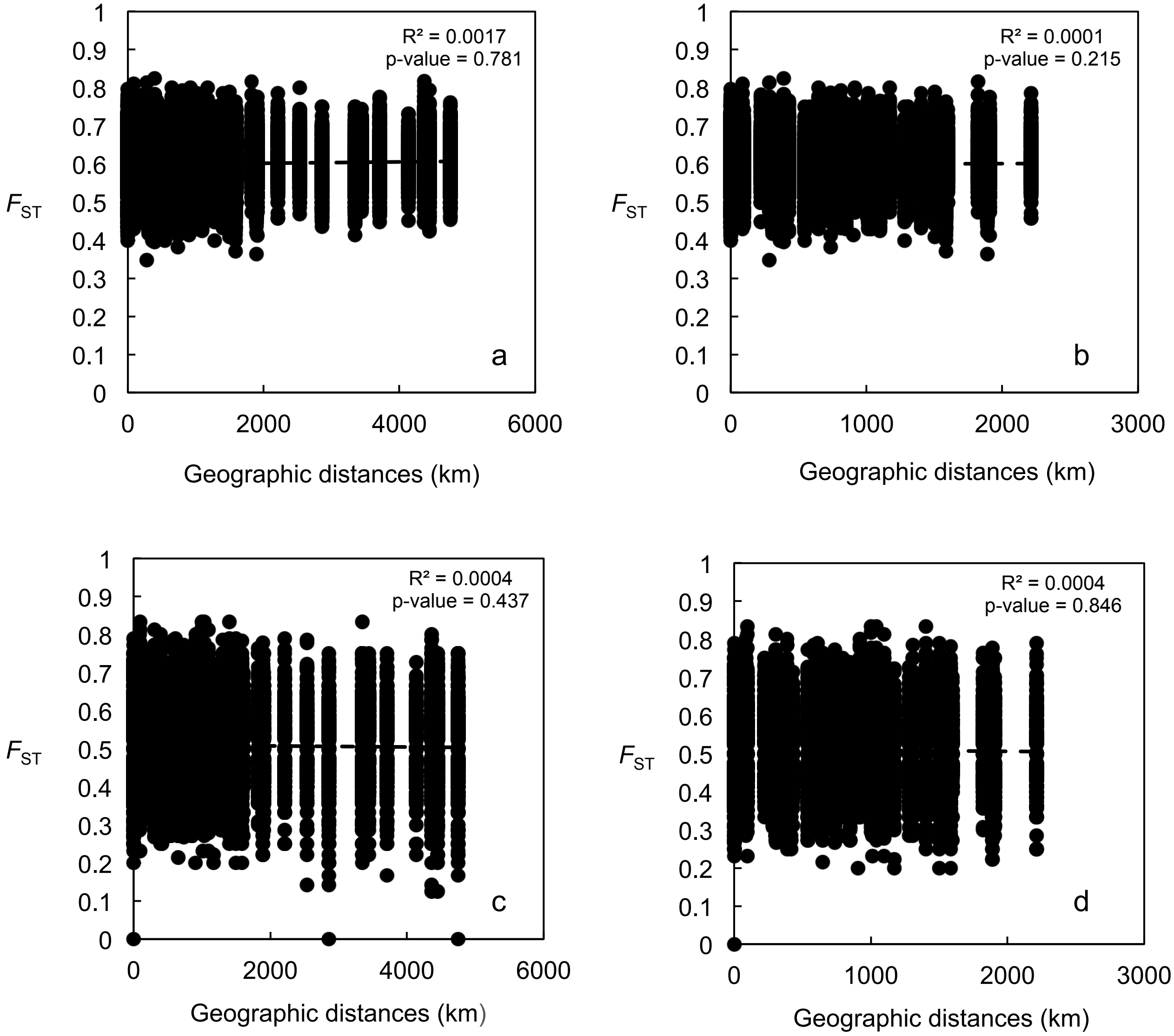

Figure SM6. IBD analyses between individuals for, **a)** 15 putatively neutral loci and the 186 US individuals, **b)** 15 putatively neutral loci and the 161 GOM individuals, **c)** US-seven-loci-dataset and the 186 US individuals, and **d)** US-seven-loci-dataset and the 161 GOM individuals
